# Unraveling the Temperature-Dependent Relaxation Dynamics of Ionic Liquid-Plasticized Compleximers

**DOI:** 10.1021/acs.macromol.5c01318

**Published:** 2025-07-11

**Authors:** Sophie G. M. van Lange, Riccardo Biella, Diane W. te Brake, Sinty Dol, Maarten Besten, Joris Sprakel, Santiago J. Garcia, Jasper van der Gucht

**Affiliations:** † Physical Chemistry and Soft Matter, 4508 Wageningen University and Research, 6708 WE Wageningen, The Netherlands; ‡ Department of Aerospace Structures and Materials, Faculty of Aerospace Engineering, 312572 Delft University of Technology, 2629 HS Delft, The Netherlands; ¶ Laboratory of Biochemistry, 4508 Wageningen University and Research, 6708 WE Wageningen, The Netherlands

## Abstract

Polyelectrolytes with ionic domains screened by bulky hydrophobic segments form processable, hydrophobic complexes called “compleximers”. Ionic liquids, which are chemically similar, further plasticize compleximers, yet the mechanisms behind their plasticization effects and distribution within the complexes remain unclear. This study examines the relaxation dynamics of plasticized compleximers across multiple length scales using rheology, fluorescence recovery after photobleaching (FRAP), and broadband dielectric spectroscopy (BDS). The incorporation of ionic liquids into compleximers reduces their glass transition temperature (*T*_g_), accelerates diffusive processes, increases segmental motion, and leads to a small decrease in activation energy associated with these relaxation processes. However, the activation energies vary substantially between techniques, probing different physical processes: approximately 200 kJ/mol in rheology, 50 kJ/mol in FRAP, and 90 kJ/mol in BDS. These variations suggest that collective dynamics strongly influence the compleximer rheology, making the mobilization (and activation) of polymer chains distinct from the local movement of ionic segments.

## Graphical abstract

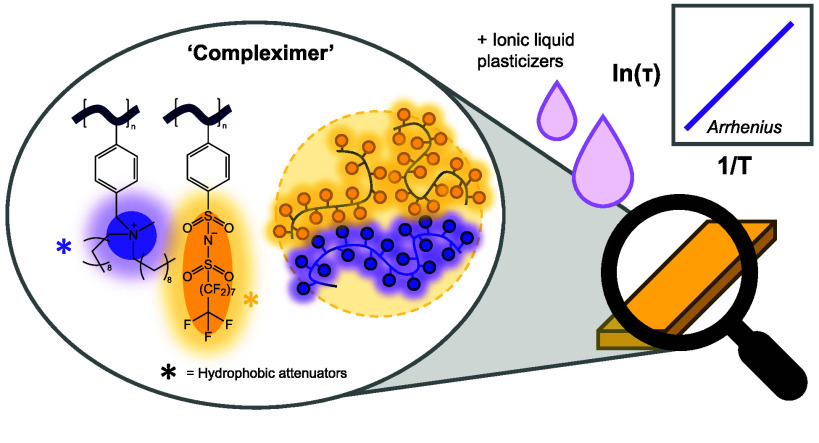

## Introduction

Nature effectively utilizes ionic interactions to structure and strengthen materials. Organisms like sandcastle worms^1^ and velvet worms,^2^ for example, have evolved to exploit the attraction between opposite charges to create underwater adhesives and biological slimes. Based on the same interactions, cellular compartmentalization in membraneless organelles in cells arises from the complexation of charged RNA and proteins.^3^ The prevalence and diversity of charged macromolecules in nature can lead to the formation of complex yet highly functional materials. Capturing this functionality in synthetic systems has remained largely limited to the formation of moderately hydrated polyelectrolyte complexes (PECs),^4^ and liquid complex coacervates^5^ from mixing solutions of (synthetic) polyelectrolytes. In nature, PECs form and operate in highly hydrated environments, where water and dissolved salts act as plasticizers by screening the electrostatic interactions.^6,7^ In the lab, it has been challenging to synthetically produce PECs that break free from their plasticized environment and, rather than being sticky or slimy, harness ionic interactions as (reversible) cross-linkers, mimicking the behavior of covalent bonds in thermosetting plastics. In addition to their sensitivity to moisture, dried PECs are extremely brittle and challenging to process due to the absence of a glass transition.^8^

We have previously shown, however, that introducing hydrophobic segments into the polyelectrolyte structure enables the creation of PECs that resist hydration, and referred to these as “compleximers”.^9^ By incorporating bulky, hydrophobic domains near charged groups, compleximers achieve an internal plasticizing effect, reducing rigidity, lowering the glass transition temperature (*T*_g_) and thus enabling processability at elevated temperatures below the degradation temperature and without water. This innovation results in a hydrophobic, physically cross-linked material that combines thermoplastic-like recyclability with the network stability of thermosets, based on ionic interactions.

These compleximers were found to exhibit a very broad thermal transition, extending to temperatures that are close to the degradation temperature of the polymers. The glass transition temperature could be lowered further by adding external plasticizers, which allowed us to capture the full transition. The bulky ions in compleximers are molecularly very similar to ionic liquids, a class of organic salts that are fluid at temperatures typically below 100 °C.^10^ We therefore found ionic liquids to be very effective plasticizers of compleximers, lowering their *T*_g_ and rendering them flexible under mild heating.^9^ Plasticization in polymeric materials typically occurs through the addition of small molecules that enhance flexibility by lubricating the polymer chains by reducing friction, disrupting intermolecular interactions, and/or increasing free volume.^11^ In PECs, the water content^6,12^ (controlled through modulating the relative humidity) and the salt concentration^13,14^ affect the energy barriers for the relaxation, which has been described previously by time-water and time–salt superpositions. The precise mechanism by which ionic liquids facilitate plasticization in water-free compleximers and how they are distributed within the compleximer matrix has remained unexplored.

In this work, we employ several experimental techniques to unveil the relaxation behavior of compleximers plasticized with ionic liquids (Schematic, Figure 1). Time–temperature superposition (TTS) applied to the rheology data obtained from oscillation experiments allows us to construct master curves of viscoelastic behavior by shifting data obtained at different temperatures, offering insights into the material’s bulk relaxation behavior. Fluorescence lifetime imaging microscopy (FLIM) and fluorescence recovery after photobleaching (FRAP) are used to study the microstructure and dynamics of the material, providing insights into the distribution and movement of small molecules within the compleximer. Broadband dielectric spectroscopy (BDS), here used in combination with TTS, probes the material’s response to an oscillating electric field, allowing us to study the frequency-dependent molecular dynamics and dielectric properties, which are closely related to the mobility of ionic and polymeric species. These techniques reveal that ionic liquids lower the material’s *T*_g_ and accelerate its dynamics, resembling a unique Arrhenius-type relaxation that has only been observed in organic vitrimer materials.^15^ Plasticization has a minimal impact on the fundamental relaxation processes in compleximers, as the activation energies change only moderately, regardless of plasticizer type or content. However, significant discrepancies in activation energies are observed between the different methods, which probe different relaxation phenomena. This suggests that in compleximers, relaxations on large scale, where cooperative effects play a role, are different from the mobility of small molecules and local movements.

**Figure 1:**
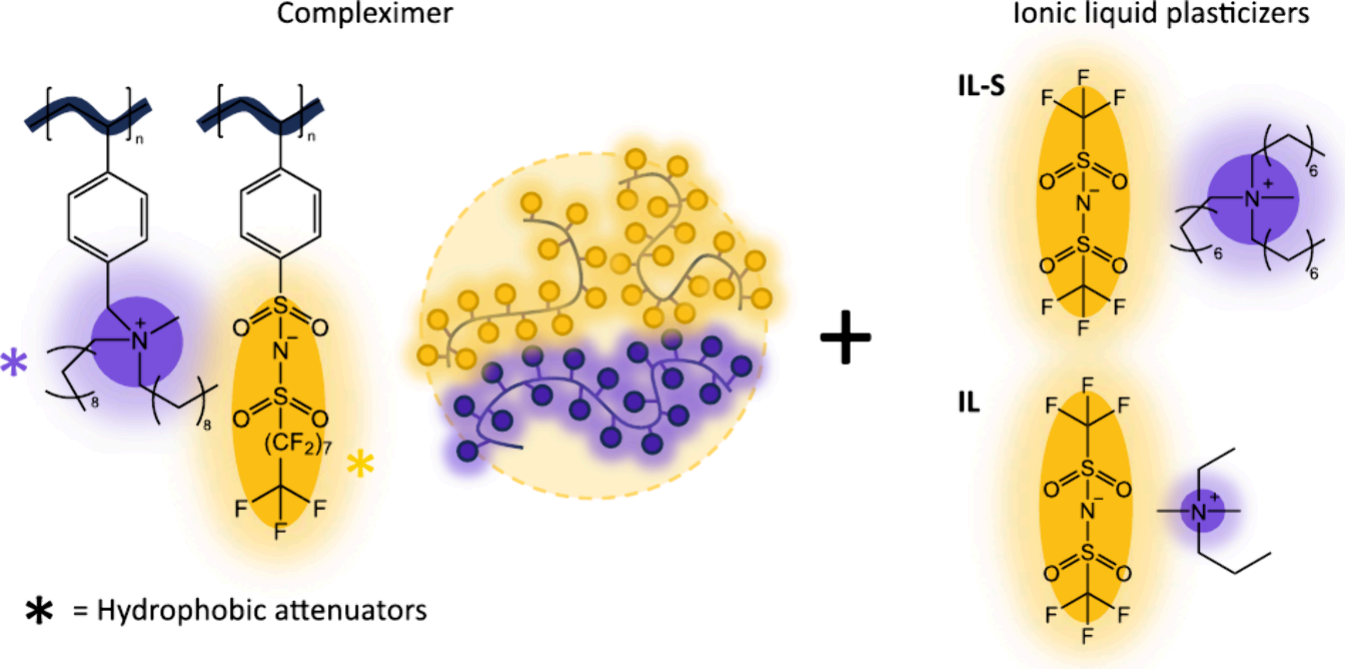
Schematic of polystyrene based compleximers plasticized with ionic liquids. IL-S is a bulky ionic liquid with a polycation screened by long hydrocarbon spacers. IL is a small molecule ionic liquid.

## Experimental Section

### Sample Preparation

The synthesis and processing of screened (S) compleximers, including the addition of plasticizers dioctyl phthalate (DOP), as well as two ionic liquids: screened methyl-trioctylammonium bis­(trifluoromethyl­sulfonyl)­imide (IL-S) and nonscreened ethyldimethyl­propylammonium bis­(trifluoromethyl­sulfonyl)­imide (IL) was described in our previous work.^9^

### Dynamic Mechanical Analysis (DMA)

We here prepared samples with dimensions 2 × 0.5 × 0.1 cm for use in extensional DMA. For DMA in shear, we prepared samples with a diameter of 1 cm and a thickness of approximately 0.1 cm. Samples with 0% ionic liquid and 5% DOP were performed in extension. Samples with 10% and 20% IL-S and 35% IL were performed in shear. Extensional moduli were converted to shear moduli assuming a Poisson ratio of 0.5, so that E = 3G, which shows excellent agreement (Figure S1). We apply the time temperature superposition (TTS) principle to frequency sweeps recorded at a range of temperatures, using an Anton Paar 702 Space Multidrive. The extensional DMA fixture was used for extensional oscillatory measurements on the rectangular compleximer samples. The round samples were measured with a 10 mm in diameter crosshatched plate–plate geometry.

#### Time Temperature Superposition (TTS)

After fixing the sample in the DMA, a temperature equilibration was performed at 170 °C for 10 min prior to the measurement to ensure full equilibration, and adhesion to the parallel plate when used. Subsequently, frequency sweeps were performed from 170 to 40 °C in steps of 10 °C. Between each measurement, the new temperature was held for 5 min to ensure proper equilibration. The frequency sweep experiments ran between 10^2^ and 10^–1^ rad s^–1^ (or 10^2^ and 10^0^ rad s^–1^ for the samples with 35% IL-S, DOP plasticized samples) while oscillating with a strain of 0.01%. In extension, a preload force of 1500 Pa was applied, and in shear, a Normal force of 2 N was applied. Mastercurves were then constructed through shifting the moduli and tanδ values horizontally by multiplying the frequency axis with a temperature dependent shift factor *a*_*T*_ relative to the reference temperature of 120 °C. The activation energies were extracted by fitting to the Arrhenius equation.

### Fluorescence Lifetime Imaging (FLIM)

#### Sample Preparation

Fluorescently labeled samples, including ionic liquids, were prepared by swelling powdered compleximer powder in dimethylformamide (DMF) containing a stock solution of Azido Bodipy^16^ and a desired amount of ionic liquid. The final Bodipy content in the dried compleximer film was calculated to be 0.001%. Upon overnight swelling, the DMF was removed with a rotary evaporator, after which the powder was further dried in a vacuum oven. When fully dried, the powder was placed between two Teflon sheets and hot-pressed at 110 °C to obtain thin compleximer films. These films were transferred to object glasses.

#### FLIM Measurements

Fluorescence lifetime imaging microscopy (FLIM) was performed on a Leica TCS SP8 inverted confocal microscope equipped with a white light laser (WLL) and a Becker & Hickl SPC830 time-correlated single photon counting (TCPC) module. The Bodipy rotor fluorophore was excited with a 488 nm laser, emission was captured between 500 and 550 nm through a 10× air objective on a hybrid detector. Acquisition times were typically between 60 and 120 s, images were obtained in a 256 × 256 pixel format. Images were processed in SPCImage 8.5 to generate two-component exponential decay curves for every pixel. FLIM images are presented in a false-color scale that represents the mean fluorescence lifetime.

### Fluorescence Recovery after Photobleaching (FRAP)

#### Sample Preparation

To prepare the samples for temperature resolved FRAP, compleximer powder was swollen in DMF, which contained fluorescent dye Pyrromethene 546 (0.02–0.03 wt %), and the ionic liquid in different concentrations based on the compleximer mass (20% for IL-S and 35% for IL). These mixtures were stirred overnight, after which the solvent was removed using a rotary evaporator at 60 °C. The FRAP samples were dried in a vacuum oven at 60 °C for at least 3 nights. The compleximers were hot-pressed into thin films using a Specac manual hydraulic press with heated plates between two Teflon sheets. The compleximer powders were heated for 15 min at 160 °C after which 1.5 tons of pressure was applied for 15 min. The samples were then removed from the press and cooled to room temperature.

#### FRAP Measurements

Temperature resolved FRAP measurements were performed on a Nikon C2 Confocal laser scanning microscope using a 488 nm (15 mW) laser for both measuring and bleaching. The resolution of the images was 512 × 512 px. A disk-shaped Region of Interest (ROI) with a diameter of 30 μm was selected for bleaching at a depth of approximately 10–15 μm. Before the bleaching, 21 images, and after bleaching, 901 images were recorded with a time interval of 2 s at a laser power of 0.15 mW. The bleach consisted of 3 s of 15 mW laser power. The temperature was controlled using a VAHEAT standard Range system from Interherence GmbH in Erlangen. A hot-pressed sample was placed on a smart substrate (SmS) standard range with dimension 18 × 18 × 0.17 (±0.05) mm^3^. A Plan Apo 10× (NA = 0.45, WD = 4000 μm) air objective was used with a digital zoom of 4x. FRAP experiments were performed at four temperatures: 25, 50, 75, and 100 °C. Before bleaching, 5 images were recorded, and after bleaching 91 images, with a time interval of 10 s between images.

#### Analyzing FRAP Data

FRAP data was analyzed using the method of Bos et al.^17^ adapted from Taylor et al.^18^ The fluorescence in the bleach spot was normalized using

$$\left\langle C\right\rangle \left(t\right)=\frac{\left\langle C\left(r,t\right)\right\rangle -\left\langle C\left(r,0\right)\right\rangle }{\left\langle C_{\mathrm{prebleach}}\right\rangle -\left\langle C\left(r,0\right)\right\rangle }$$ 1

where ⟨*C*(*r*, *t*)⟩ is the average intensity of fluorescence in the bleach spot during the measurement, and ⟨*C*(*r*, 0)⟩ is the average intensity of fluorescence in the bleach spot immediately after bleaching. ⟨*C*_prebleach_⟩ represents the average intensity in the bleach spot before bleaching. This data was then corrected for changes in background fluorescence during the measurements.

$$C_{\mathrm{cor}}\left(t\right)=\left\langle C\left(t\right)\right\rangle \frac{C_{\mathrm{ref}}\left(0\right)}{C_{\mathrm{ref}}\left(t\right)}$$ 2

where *C*_cor_ is the corrected intensity concentration. ⟨*C*⟩ is the normalized intensity in the bleach spot. *C*_ref_(0) is the intensity in the reference spot far from the bleach spot in the image immediately after bleaching, while *C*_ref_(*t*) is the intensity in the reference spot during the measurement.

### Broadband Dielectric Spectroscopy (BDS)

Measurements were performed on a Novocontrol BDS Alpha analyzer with Quatro temperature control system. N_2_ gas was used for the temperature control. Disk-shaped samples with 10 mm diameter, and 1 mm thickness were used for the experiments. Prior to the experiments, samples were coated with 15 nm of gold using a Q300TD Sputter coater to improve the electrical contact. The samples were mounted between brass electrodes with a diameter of 10 mm. The samples were dried in the sample setup for 1 h at 80 °C. After the drying process, the electrical contact was further tightened ensuring proper electrical contact. The tests were carried out between 10^6^–10^–1^ Hz and 160–0 °C at a root-mean-square voltage of 1 V.

## Results and Discussion

### Ionic Liquids Plasticize Compleximers without Affecting the Apparent Activation Energy

We apply the Time Temperature Superposition (TTS) principle to compleximers plasticized with ionic liquids (the bulky ionic liquid methyl-trioctylammonium bis­(trifluoromethyl­sulfonyl)­imide (IL-S) and the smaller ethyl-dimethylpropylammonium bis­(trifluoromethyl­sulfonyl)­imide (IL)). Here, we focus on ionic liquid concentrations that give a *T*_g_ that is low enough to capture the full thermal transition (Table S1). The TTS principle is based on the idea that increasing the temperature of a material has a similar effect as reducing the time over which it is deformed.^19^ This occurs because the different relaxation times associated with a particular relaxation process share the same temperature dependence.^20^ Using this principle, we can probe the material’s mechanical response at time scales that are otherwise inaccessible, either due to instrument limitations or experiment duration limitations.^21^ To this end, we measure a series of frequency sweeps at a range of temperatures for screened polystyrene compleximer (Figure 1) samples plasticized with 0%, 10% and 20% screened ionic liquid IL-S (Figure 1), which is a bulky ionic liquid that resembles the chemical structure of the compleximer. The storage and loss moduli, reported in Figure 2A-B, D-E and G-H, show an overall decrease with increasing temperature, indicating a temperature-induced softening of the material. Yet, at all explored temperatures, the compleximers remain solid, as the storage moduli are always larger than the loss moduli. The tan δ $\left(\tan\delta =\frac{G^{\prime \prime }}{G^{\prime }}\right)$ values, represented in Figure S2 are thus always <1 in the employed frequency range. We verify the rheological simplicity of the samples by constructing Van Gurp-Palmen plots,^22^ plotting the phase shift as a function of the complex modulus (Figure S3). These curves are expected to merge onto a single line when TTS is applicable; we note that TTS holds well for most samples, though deviate somewhat from rheological simplicity with high ionic liquid concentrations. Master curves are constructed from these data by shifting the curves horizontally relative to a reference temperature of 120 °C by multiplying the frequency axis with only a temperature-dependent shift factor *a*_*T*_ (Figure 2C, F and I respectively), no vertical shift factors were used. This shifting gives access to the relaxation spectrum over more than ten decades of frequency. The resulting relaxation spectra appear notably broad, with extended power law regimes at low frequencies, with slopes of approximately 0.1. We observe a shift in the dynamic glass transition, which can be identified by the peaks, here the very broad shoulders, in the loss modulus and tanδ master curves. Ionic liquids clearly lower the glass transition temperature (*T*_g_), also known as the α transition,^23,24^ as these features shift to higher frequencies. This aligns with our findings on the *T*_g_ of these systems, as measured from temperature sweeps, presented in our previous work^9^ Table S1 and Figure S4.

**Figure 2:**
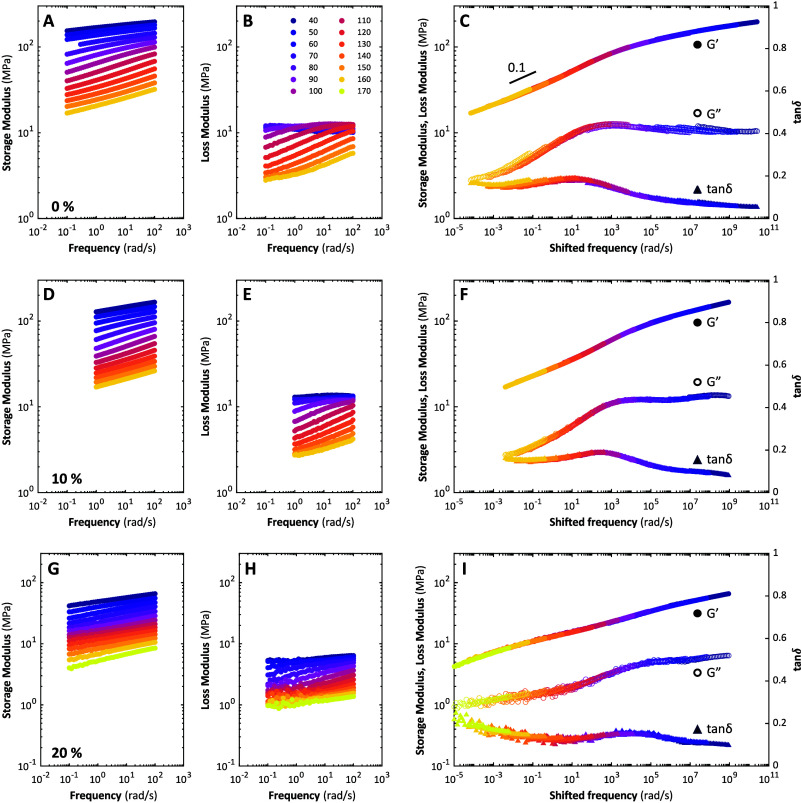
Time temperature superposition (TTS) of compleximer with increasing concentration of ionic liquid (IL-S). (A) The storage modulus (*G′*) and (B) loss modulus (*G*″) of nonplasticized compleximer are shifted horizontally with respect to a reference temperature (*T*_*ref*_) of 120 °C to form a (C) master curve. Vertical shift factors were not needed. The same is done for the (D–F) compleximer plasticized with 10% IL-S and (G–I) compleximer plasticized with 20% IL-S.

When shifting TTS master curves with different plasticizers and plasticizer content onto one single master curve relative to the nonplasticized compleximer, we can identify a time–temperature-plasticizer superposition (Figure 3). In case of plasticizing the compleximer with (small amounts) of plasticizers IL-S and a neutral plasticizer dioctyl phthalate (DOP), the dynamical relaxation mechanisms are not fundamentally changed but accelerated by orders of magnitude, as shown in the shift factors (Figure 3, caption), as they collapse on the same master curve. A larger amount (35%) of a nonscreened, small molecule plasticizer IL (Figure 1) leads to deviations from this master curve, likely by changing the relaxation mechanism. In our previous work, SAXS analysis revealed that this concentration of IL alters the network structure,^9^ potentially accounting for the differences in relaxation.

**Figure 3:**
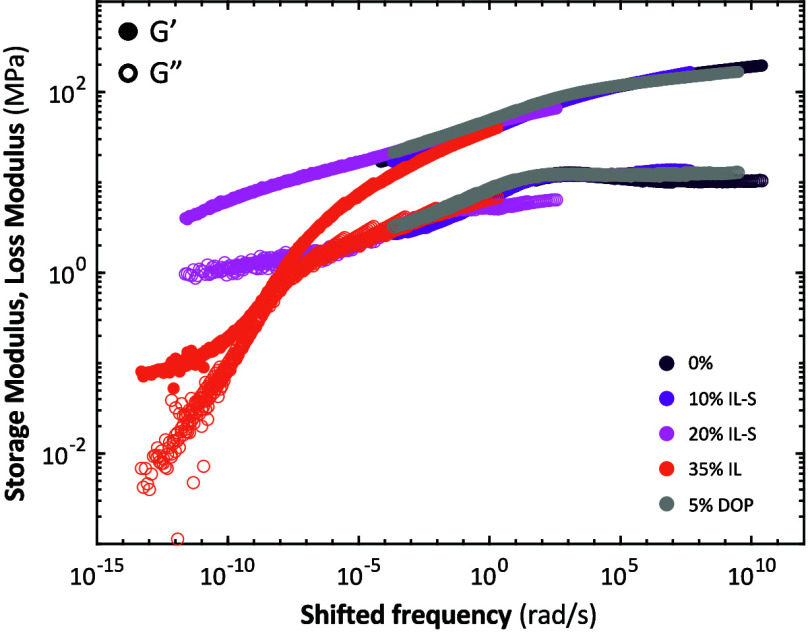
Time–temperature-plasticizer superposition of plasticized compleximers. The master curves are shifted along the frequency axis relative to the 0% data until the largest portion of the curves overlap, forming a single master curve.. The respective shift factors for 10% IL-S, 20% IL-S, 35% IL, and 5% DOP were 0.05, 4 × 10^–7^, 1 × 10^–10^, and 0.7.

Above *T*_g_, strong glass-forming materials such as silica glass^25^ and “vitrimers” ^15,26^ —dynamic polymer networks that can undergo bond exchange reactions—show a gradual trend in the relaxation time (which scales with the shift factor *a*_*T*_), characterized by a constant activation energy that complies with the Arrhenius law:

$$\ln\left(a_{T}\right)=\ln\left(A\right)+\frac{E_{a}}{R}\cdot \frac{1}{T}$$ 3

where *A* is the pre-exponential factor, *E*_*a*_ the activation energy in J/mol and *R* the gas constant. Most glassy polymers deviate strongly from this trend and show a much steeper decrease in the relaxation time above *T*_g_, which can be described empirically by the Williams–Landel–Ferry (WLF) model:^27,28^

$$\log\left(a_{T}\right)=-\frac{C_{1}\left(T-T_{\mathrm{ref}}\right)}{C_{2}+\left(T-T_{\mathrm{ref}}\right)}$$ 4

where *C*_1_ and *C*_2_ are constants, and *T*_*ref*_ is the reference temperature with respect to which the curves are shifted. We observe in Figure 4 that IL-S plasticized compleximers in fact follow an Arrhenius law over the entire temperature window, thus exhibiting behavior similar to that of vitrimers. The activation energies of the plasticized samples are slightly lower than the nonplasticized one, but do not decrease further upon adding more ionic liquid. Plasticization with 35% of a nonscreened ionic liquid IL again leads to a small decrease of the activation energy (Figure S6), even though the moduli are much more strongly affected, as shown in our previous work.^9^ Finally, plasticization with 5% of a nonionic plasticizer DOP also leads to a similar activation energy (Figure S7). We note that accurate determination of shift factors - and therefore activation energies - is challenging at temperatures below *T*_g_. For the samples where this is possible, we have therefore determined the activation energies using data above *T*_g_ only. For the pure compleximer, where *T*_g_ is too high, we report an activation energy using data below *T*_g_. We note, however, that for the samples with lower *T*_g_ differences in apparent activation energy below and above *T*_g_ are modest. All activation energies found are in the range of 200–250 kJ/mol, which is very high compared to the theoretically expected bond energy of an ionic bond according to Coulomb’s Law, which is in the order of 50 kJ/mol (Note S1). In Note S2, we explain how the “apparent activation energy” can be significantly higher than the true *E*_*a*_ when *E*_*a*_ itself depends on temperature,^29^ which may be the case here, and also demonstrate that when *E*_*a*_ varies linearly with *T*, the Arrhenius plot remains a straight line. Clearly, all plasticizers accelerate the relaxation, but with small impact on its temperature dependence. This behavior is particularly noteworthy given that ionic plasticizers in hydrated polyelectrolyte complexes typically reduce the activation energy more strongly,^30,31^ suggesting a different mechanism is at play here.

**Figure 4:**
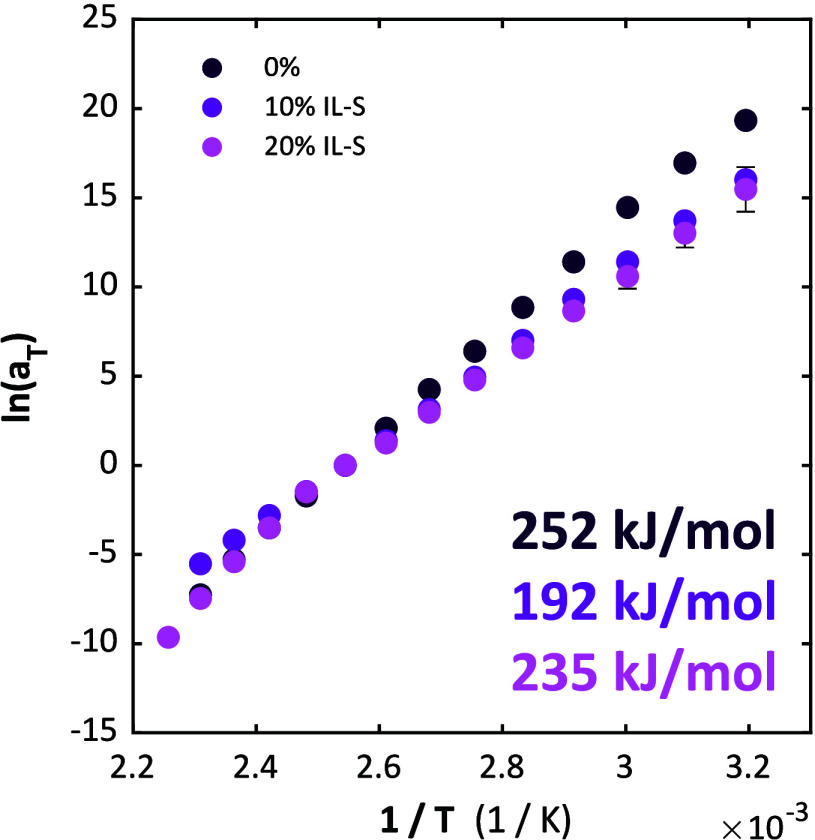
Arrhenius plots and activation energies of compleximers with 0% IL-S, 10% IL-S, and 20% IL-S. All three samples follow the Arrhenius law and have similar activation energies. All shift factors were determined relative to a reference temperature of 120 °C.

### Ionic Liquids Enhance the Diffusion of Small Molecules in Compleximers

With rheometry, macroscopic relaxation of the material as a whole is probed. Using rheological TTS we find activation energies for structural relaxation to be much higher than those expected for the molecular relaxation of ionic supramolecular interactions. This could suggest that in rheometry collective relaxations taking place at larger length scales are probed. To investigate the material dynamics at smaller length scales, where collective effects are expected to be smaller, we study the diffusion of small molecules in the polymer matrix, using fluorescence recovery after photobleaching (FRAP). This method is typically used to investigate diffusion in biological cells,^32^ but has also been used to study mobility in polyelectrolyte multilayers.^33,34^ For this technique, we incorporate a fluorescent dye, Pyrromethene 546, in addition to plasticizers, in compleximer films which we investigate under the confocal microscope. The diffusion of the fluorescent dye in a polymer matrix can be interpreted using theories based on free volume. The thermal expansion of the material leads to an increase in free volume and a concomitant increase of the diffusion coefficient.^35,36^ We bleach a disk-shaped region of interest (ROI) in the compleximer, and study the recovery of the fluorescence intensity, resulting from diffusive processes, as a function of time. Due to diffusive mixing, unbleached fluorophores mix with the bleached fluorophores in the bleach spot, leading to an increase of fluorescence over time. As the acquisition of the images used to map recovery can also induce mild bleaching, we correct for this by recording background intensities in each frame (Figure S8). We measure the recovery at four temperatures: 25, 50, 75, and 100 °C for S compleximers with 0% ionic liquid (Figure S9), 20% IL-S (Figure S10) and 35% IL (Figure S11). We measure at least 8 different spots per temperature for each of the samples, and plot the average recovery curves in Figure 5A–C.

**Figure 5:**
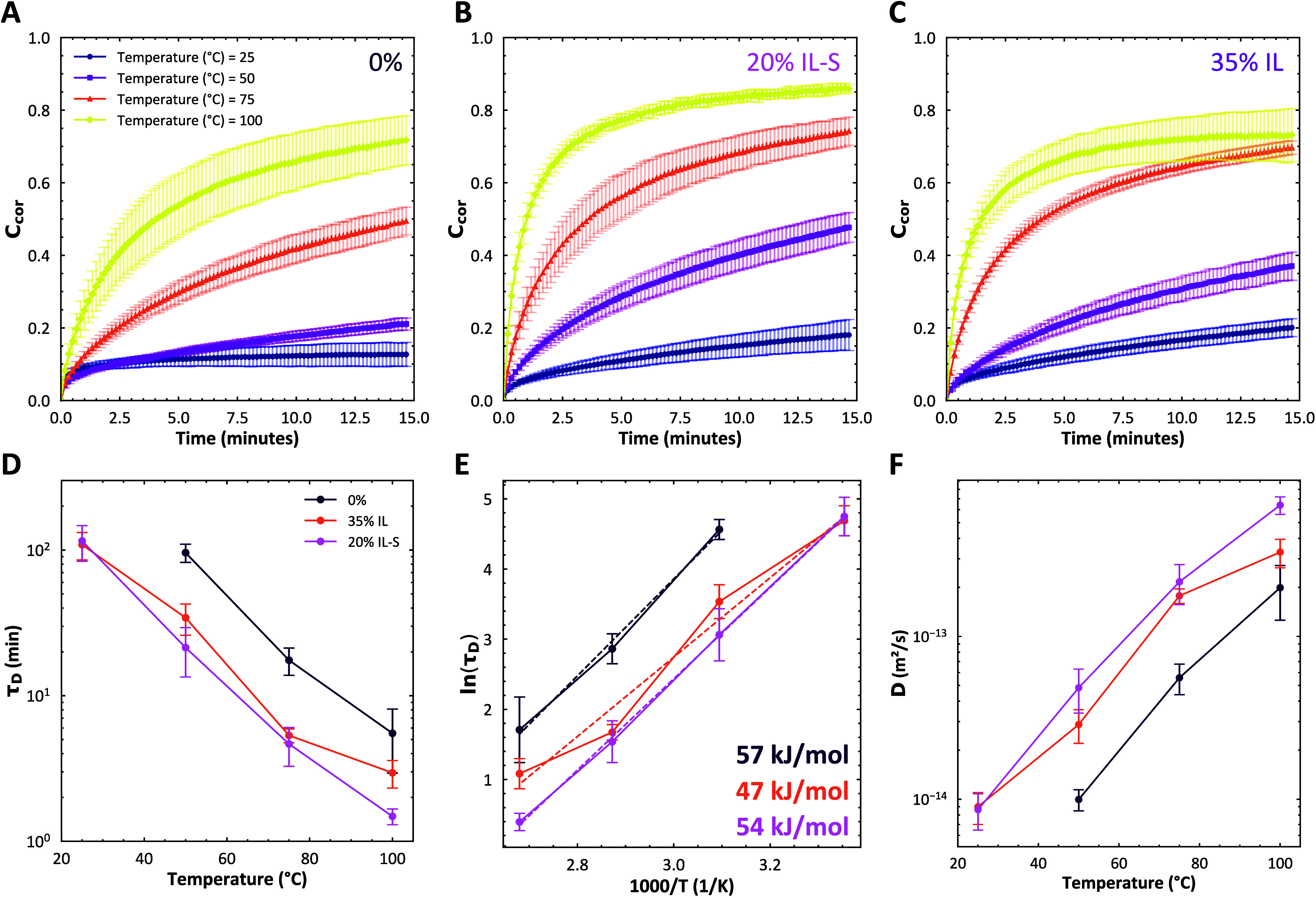
The fluorescence recovery after photobleaching of compleximers at elevated temperatures. The intensity was corrected with respect to a reference spot. The corrected intensity (*C*_*cor*_) was here plotted as a function of time for (A) 0% IL-S, (B) 20% IL-S, and (C) 35% IL. The errors are represented by the shaded areas. In all cases, the recovery is accelerated with increasing temperature. The recovery curves were fitted with a 2D diffusion model for a disk-shaped bleach spot. (D) The characteristic relaxation time τ_*D*_ of the diffusion model as a function of temperature shows a decrease in the relaxation time with temperature. Ionic liquid plasticizers speed up the relaxation. (E) The Arrhenius plot shows that the relaxation times roughly follow an Arrhenius law with activation energies around 50 kJ/mol. (F) The diffusion coefficients were calculated from the diffusion times using eq 6.

The fluorescence recovery is generally accelerated with increasing temperature. At 25 °C, the initial recovery is relatively fast, which may be due to temporary heating of the sample as a result of the high-intensity laser. We also show through plotting the corrected recovery at a fixed time point, 10 min, as a function of temperature, that the recovery is accelerated with temperature and with the addition of ionic liquid. The screened ionic liquid IL-S, which has a bulkier cation, is slightly more effective than the nonscreened IL (Figure S12).

The more subtle effects of temperature and plasticizer content can be more effectively quantified by fitting the individual recovery curves with a diffusion model for disk-shaped ROIs:^37,38^

$$f\left(t\right)=\exp\left(-\frac{2\tau_{D}}{t}\right)\left[I_{0}\left(\frac{2\tau_{D}}{t}\right)+I_{1}\left(\frac{2\tau_{D}}{t}\right)\right]$$ 5

where *I*_0_ and *I*_1_ are modified Bessel functions of the first kind, τ_*D*_ is the characteristic diffusion time and the maximum recovery is set to 1. The diffusion coefficient *D* can be calculated through

$$\tau_{D}=\frac{r^{2}}{4D}$$ 6

with *r* the radius of the photobleached area. Despite having only one fitting parameter, the model fits the recovery data well, except for the nonplasticized compleximer at 25 °C, where the heating of the sample during bleaching interferes with the recovery process. For all other samples, we find that the characteristic diffusion time τ_*D*_ decreases with increasing temperature, but also that the addition of plasticizer overall lowers τ_*D*_ somewhat (Figure 5D). This means that the ionic liquid makes the polymer matrix more mobile or that the fluorescent dye can diffuse through the ionic liquid to the bleached spot. The Arrhenius plot of the FRAP diffusion times gives activation energies around 50 kJ/mol for the plasticized samples, much smaller than found from the rheology data (Figure 5E). This suggests that the relaxation of the matrix at the molecular scale, as probed through small molecule diffusion, is governed by a different processes than that found in the macroscopic rheology experiments above. An overview of the relaxation times of the various samples is reported in Table S2. The corresponding diffusion coefficients *D* are roughly between 10^–14^ and 10^–12^ m^2^/s (Figure 5F). These diffusion coefficients are relatively high compared to those for, for example, small molecules in PMMA in the vicinity of *T*_g_.^39^ This suggests that the free volume is larger in our compleximers than that in traditional thermoplastics.

### Plasticized Compleximers Are Heterogeneous

Given that the macroscopic and molecular measurements of material dynamics suggest that different processes influence relaxation at different length scales, we asked the question about the spatial organization of the material. Are the polymer and plasticizer homogeneously distributed throughout the material, or are there distinct spatial heterogeneities that could explain the length-scale dependent relaxation? Here we map the spatial distribution of material dynamics using molecular rotor dyes: a class of fluorescent dyes whose fluorescence lifetime is sensitive to the degree of free volume in their immediate surroundings.^16^ Using Fluorescence Lifetime Imaging (FLIM), this gives access to free volume maps of the material at confocal (∼200 nm) resolution. In low-viscosity environments, or when the environment allows free rotation, this dye loses its energy through nonradiative processes (without emitting light), leading to a short fluorescence lifetime. In more viscous environments, or when rotation is hindered, the dye releases the energy via fluorescence (light emission), resulting in a longer lifetime.^40^ We here make compleximer films with both rotor dye and ionic liquid incorporated in a range of concentrations and measure the fluorescence lifetimes at room temperature (Figure 6A for the unplasticized material, B–D for the IL plasticizer and E–G for the IL-S plasticizer). Note that the films fragmented upon transferring them from the hot press to the microscope slide, so that these measurements were done on flakes of compleximer samples, rather than on bulk films. We can nevertheless interpret these data qualitatively, and observe that compleximers are highly heterogeneous; the lifetimes vary from region to region within a single flake. Distinguishing whether the heterogeneity arises from intrinsic variations in the material or from an uneven distribution of the ionic liquid is challenging and we note that the sample with 0% plasticizer also shows considerable heterogeneity. However, the well-defined red spots in samples with high plasticizer content suggest the presence of domains with shorter lifetimes and, consequently, lower viscosity. These domains may represent droplets of ionic liquid that are not fully integrated into the polymer matrix. Indeed, SAXS measurements carried out previously showed a slight modulation of the scattering curve at these high ionic liquid contents, suggesting the formation of phase-separated domains.^9^ The effect of the ionic liquid content on the fluorescence lifetime and heterogeneity is not obvious from the images. We plot histograms describing the lifetime distribution in Figure S13 and find that although there is no conclusive trend in the average lifetime with increasing plasticizer content, the lowest lifetimes are better represented in samples with high plasticizer content (35% IL and 20% IL-S).

**Figure 6:**
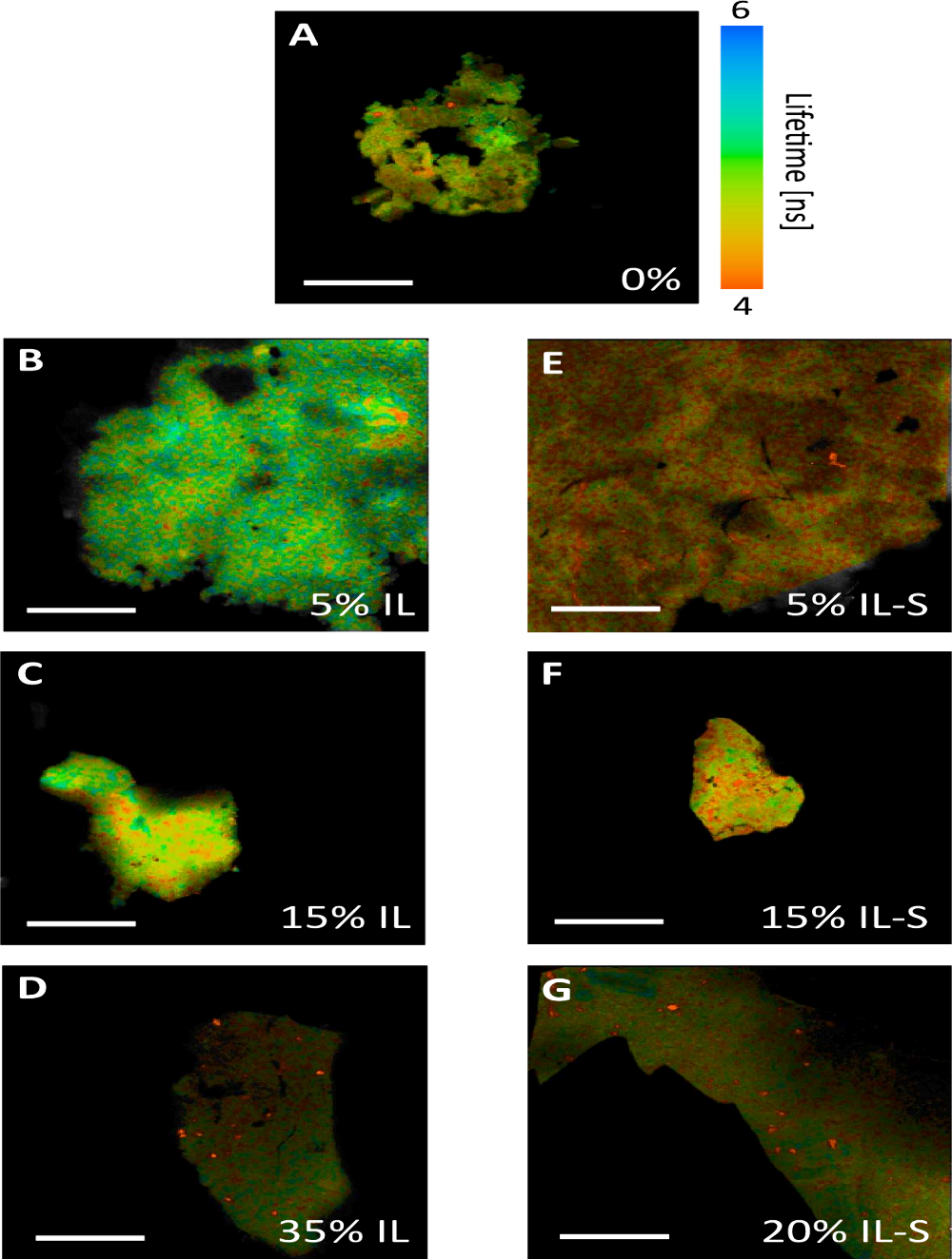
Plasticized compleximers are heterogeneous. We investigate the spatial distribution of the fluorescence lifetime using fluorescence lifetime imaging (FLIM) for compleximer flakes with (A) 0% plasticizer, with (B–D) 5, 15, and 35% IL, and with (E–G) 5, 15, and 20% IL-S. All scale bars are 300 μm. In the color bar, long lifetimes are represented in blue and short lifetimes are represented in red.

### Dielectric Relaxations in Compleximers

Since rheology and FRAP reveal markedly different activation energies due to their length-scale-dependent relaxation mechanisms, we employ broadband dielectric spectroscopy (BDS) to investigate relaxation spectra across an extensive frequency range. This approach enables the characterization of relaxations spanning a broad spectrum of length and time scales. It probes the movement of electronic dipoles resulting from an applied electric field.^41^ From dielectric experiments, we measure conductivity effects and dipole relaxation processes. Besides the α relaxation, which is a slow relaxation resulting from the cooperative movement of large segments of the polymer chains, such as the backbone or entire chain segments,^42^ ionic materials can exhibit an α_2_ relaxation. This α_2_ relaxation is found in ionomers—materials where ionic groups form clusters that restrict the mobility of the chains—showing a delayed stress relaxation.^43^ The dissociation and association of ions, or so-called “ion hopping” activates a dielectric “α_2_” process that is related to the association energy of the ionic group.^44,45^ BDS measurements of plasticized ionomers generally display an increase in the conductivity while preserving the ion transport mechanism.^46^

We perform BDS experiments on samples with 0, 10 and 20% IL-S and investigate the real part (ε′) and the imaginary part (ε″) of the dielectric permittivity. The real component, ε′ (Figure S14A, D, G), represents the material’s ability to store electrical energy by polarizing in response to the applied field.^47^ This is often influenced by dipolar orientation within the material and changes with ionic content and molecular structure.^48^ The imaginary component, ε″ (Figure S14B, E, H), relates to the energy dissipated as heat due to relaxation and conduction processes within the material.^41^ Since ε″ is susceptible to electrode polarization effects, we derive ε″_KK_ from ε′ through the Kramers–Kronig transformation, which better highlights the material relaxation effects^41,49^ (Figure S14C, F, I), following:

$$\varepsilon_{KK}^{\prime \prime }=-\frac{\pi }{2}\frac{\partial \varepsilon^{\prime }}{\partial \ln\left(\omega \right)}$$ 7

Similar to the rheological frequency sweeps, we then apply the time temperature superposition (TTS) principle to the BDS data by shifting ε′ and ε_*KK*_″ horizontally over the frequency axis by multiplying it with a temperature dependent shift factor *a*_*T*_ (Figure 7A–C for the real part and D–F for the imaginary part) with the same *T*_*ref*_ of 120 °C. These master curves show a contribution of the conductivity at low frequencies (dotted line), and a dipole relaxation at higher frequencies in the form of a broad peak or shoulder (dashed line), which shifts to higher frequencies with increasing ionic liquid concentration. These peaks are found at similar frequencies as the loss peak in the respective rheological TTS master curves (Figure 2). The fact that the permittivities collapse so well on a master curve indicates that both the conductivity and relaxation depend on temperature in the same way. The Arrhenius plot of the shift factors shows an Arrhenius dependence, with activation energies around 110 kJ/mol, displaying a very slight decrease with increasing plasticizer content (Figure 7 insets). The magnitude of the activation energies is in between those found with rheology and FRAP.

**Figure 7:**
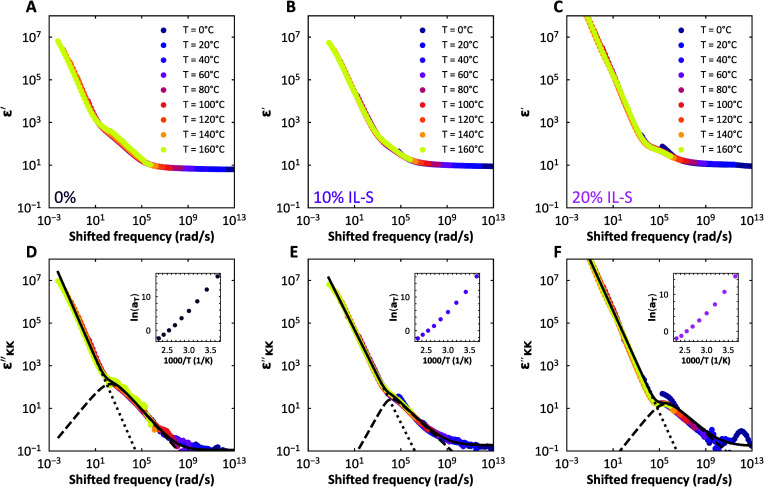
BDS master curves of compleximers plasticized with IL-S. (A, B, C), Master curve of the real part of the dielectric permittivity ε′ of S compleximer with 0%, 10%, and 20% IL-S, respectively, obtained after time–temperature superposition (TTS) with a reference temperature of 120 °C. (D, E, F), Master curve of the imaginary part of the dielectric permittivity, found through transforming ε′ using the Kramers–Kronig transformation. The master curves were fitted with a conductivity term (dotted line) and the Hraviliak–Nagami model (dashed line); the full fit is shown as a black line. The insets show the Arrhenius plots of the temperature dependent shift factors α_T_ of the TTS shifting of ε_*KK*_″ at different concentrations of ionic liquid, and the respective activation energies are 114, 83, and 78 kJ/mol. Also here, vertical shift factors were not needed.

The ε″_KK_ master curves (Figure 7) were fitted with a conductivity term at low frequencies and the Havriliak–Negami model at high frequencies. The conductivity term is described by

$$\varepsilon^{\prime \prime }\left(\omega \right)={\left(\frac{\sigma_{DC}}{\varepsilon_{0}\omega }\right)}^{n}$$ 8

where σ_*DC*_ is the direct current (DC) conductivity of the material.^41,48,50^ It indicates how easily an electric current can flow through the material under a steady-state electric field. The DC conductivity can be influenced by factors such as temperature, ion mobility, and the concentration of charge carriers.^51^ σ_*DC*_ increases with almost 2 orders of magnitude with increasing plasticizer content (Figure 8A), indicating a strong increase in ion mobility. Ionic liquids have a much higher mobility than the polymers since the ions are much smaller. We also plot *n*, which describes the frequency dependence of the imaginary part of the permittivity in the low frequency regime (Figure 8B).^52^ For pure Ohmic conductivity, *n* = 1, but the exponent may deviate due to interference of electrode polarization effects that can also show up at low frequencies. Electrode polarization is the accumulation of charges at the electrode. We here find that this coefficient is generally only slightly larger than 1, which means we mostly measure conductivity.^52^

**Figure 8:**
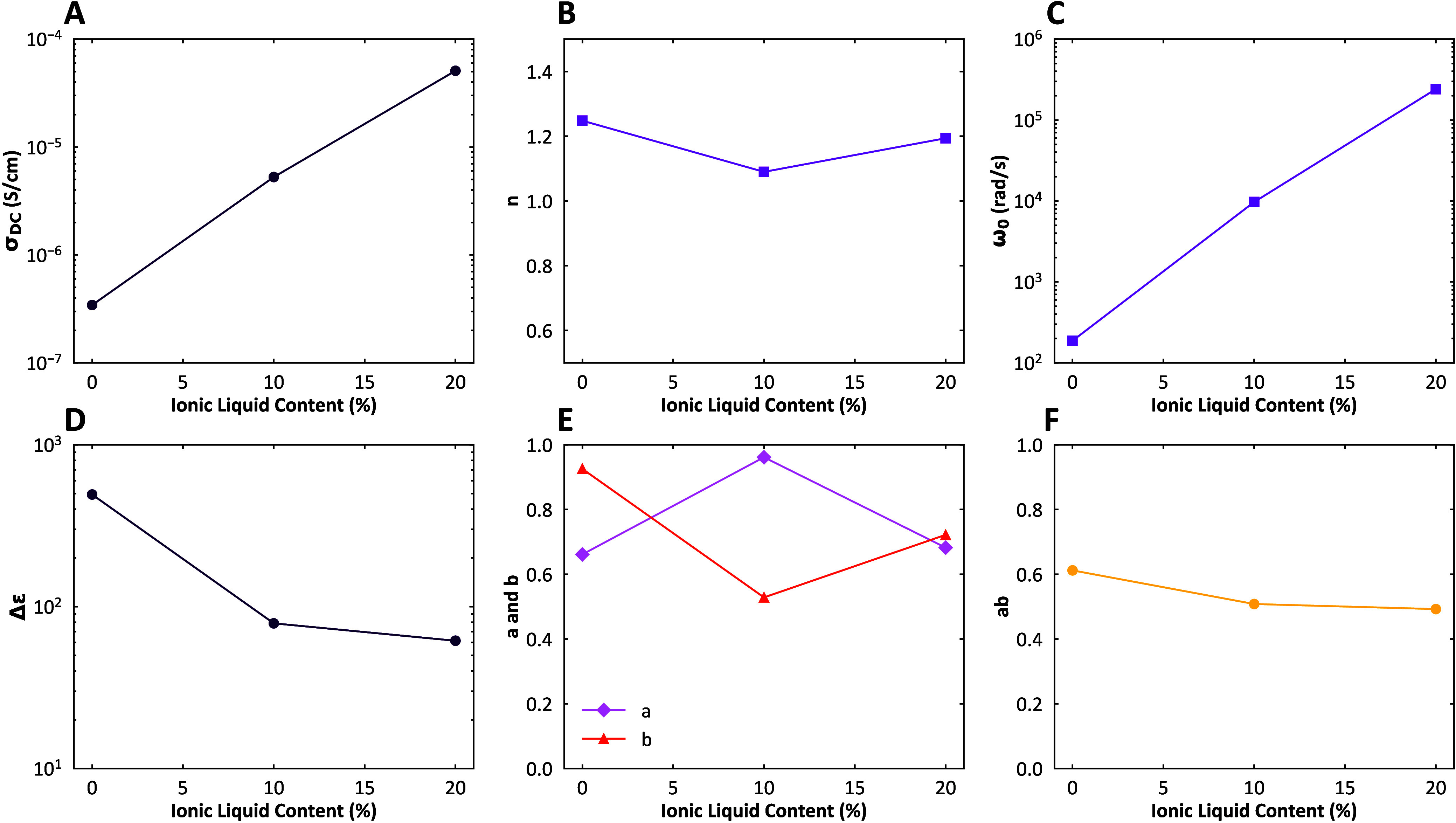
Fitting parameters of the conductivity model and Havriliak–Negami (HN) model at different ionic liquid (IL-S) concentrations. (A) σ_*DC*_, the direct current (DC) conductivity at *T*_*r*_ *ef* = 120 °C increases with ionic liquid. (B) *n* ≈ 1 which means we mostly measure conductivity effects. (C) The characteristic relaxation frequency ω_0_ increases with plasticizer content, (D) Δ*ε*, which relates to the dielectric constant, decreases. (E) *a* and *b*, which dictate the broadening and asymmetry of the relaxation peak, are similar. (F) The product of *a* and *b* is approximately 0.5.

At higher frequencies we use the Havriliak–Negami^53^ model to fit the dielectric (segmental) relaxations:

ϵHN″(ω)=Im{Δε[1+(iωω0)a]b} 9

The HN model is an (empirical) generalization of the Debye model for dipole relaxation to a broader distribution of relaxation times.^41,54^ This model is used to approximate the stretched exponential decay of the *ε*”. The parameters *a* and *b* respectively provide the broadening and asymmetry of the relaxation spectrum,^55^ where broadening is thought to be caused by dynamic heterogeneity.^41^ The peaks in the HN fits, captured by the characteristic relaxation frequency ω_0_, shift to higher frequencies with increasing temperature (Figure 8C), signifying an acceleration of the segmental motion.^56^ We also plot Δε, which represents the change in permittivity (dielectric constant) of the material (Figure 8D). Δε decreases with increasing ionic liquid plasticization. Since this parameter is related to the strength of the relaxation,^57^ we speculate that ionic liquids weaken the dipoles by being close to the ion pairs. The parameters *a* and *b* obtained from the fits are shown in Figure 8E. However, we note that *a* and *b* are difficult to fit individually since the left side of the relaxation is overshadowed by the conductivity term. The product of *a* and *b* obtained from the fits is more robust, as it is equal to the slope on the right side of the relaxation peak. This product is approximately the same for all three plasticizer contents and equal to 0.5 (Figure 8F). This may indicate that the underlying relaxation mechanisms are not affected by the ionic liquid, only accelerated. This can also be observed from Figure 7: ω_0_ and σ_*DC*_ increase with increasing temperature, but the parameters Δε, *a*, *b*, and *n* do not, as the shapes of the curves stay the same.

## Conclusions

In nature, polyelectrolyte complexes and complex coacervates such as biological adhesives and slimes typically contain hydration water and counterions, which plasticize the material. In contrast, compleximers, which are not plasticized by default, can be plasticized on demand by incorporating ionic liquids that resemble the chemical structure of the compleximer ions. While it is evident that ionic liquids effectively plasticize compleximers by lowering their *T*_g_, it has remained unclear how these plasticizers affect the material dynamics. In this study, we investigated the relaxation dynamics of compleximers plasticized with varying concentrations of two types of ionic liquids: methyl-trioctylammonium bis­(trifluoromethyl­sulfonyl)­imide (IL-S) and ethyldimethylpropylammonium bis­(trifluoromethyl­sulfonyl)­imide (IL). To probe different length scales—from the bulk material to individual ionic segments—we employed a combination of rheological time–temperature superposition (TTS), fluorescence recovery after photobleaching (FRAP), and broadband dielectric spectroscopy (BDS) combined with TTS.

Our findings demonstrate that ionic liquids effectively lower the glass transition temperature and modulus of compleximers while enhancing diffusion processes and segmental motion. Notably, the temperature dependence of these processes is not significantly affected, as the presence or concentration of ionic liquid decreases the apparent activation energy only slightly across the samples. This sets compleximer plasticization apart from conventional materials, including PECs, where the addition of a plasticizer typically results in a stronger decrease in activation energy.^30,31,58–60^ For moderately plasticized compleximers, we can identify a time–temperature-plasticizer superposition through rheological TTS. Deviations from this master curve were found when plasticizing with 35% IL, a small molecule ionic liquid, in which we also found a change in structure using SAXS.^9^ The addition of IL-S, which more closely resembles the bulkiness and chemistry of the compleximer, instead has a similar relaxation mechanism as the non plasticized compleximer. Adding neutral plasticizers dioctyl phthalate in small concentrations (5%) plasticizes compleximers, but increasing the concentration leads to phase separation^9^).

The relaxation of all compleximer samples follows the Arrhenius relation rather well, which implies that they classify as “strong glass formers”, similarly to vitrimers.^15,26^ However, the apparent activation energy of compleximers as measured with rheology is significantly higher than that of vitrimers. While the precise origin of this high activation energy is not clear, we speculate that it is due to the nature of the bonds in the material. Vitrimer relaxation is governed by exchange of dynamic covalent bonds, with a well-defined bond energy. Compleximers, by contrast, are held together by noncovalent and long-ranged ionic bonds. Upon increasing the temperature, thermal expansion of the material may thus increase the distance between the charges, thereby leading to a gradual weakening of the ionic interactions and a decrease of the activation energy. As shown previously^29^ and explained in Note S2, such a temperature-dependent activation energy can significantly increase the apparent activation energy measured with rheology.

It is important to note that the apparent activation energy derived from rheological measurements (approximately 200–250 kJ/mol) is substantially higher than that obtained from the other techniques, around 90 kJ/mol for BDS and 50 kJ/mol for FRAP (Table S2). Since the different techniques give different activation energies, this suggests they probe different relaxation processes. BDS is sensitive to the dynamics and reorientation of dipoles and ion pairs, which is governed mostly by localized segmental motions. By contrast, rheology probes viscoelastic relaxations that involve cooperative chain motion over longer length scales and reorganization of multiple ion pairs. Such cooperative motions have been shown to depend more strongly on temperature, therefore leading to a larger apparent activation energy.^61–63^ The FRAP measurements probe the diffusive motion of a neutral dye molecule in the material. This motion depends on the free volume in the material, which increases with temperature as a result of thermal expansion. However, it is probably not strongly influenced by the ionic bonds directly, which could explain the lower apparent activation energy measured with FRAP.

Despite the fact that ionic liquids have only a small effect on the activation energies, we do find they decrease the modulus at elevated temperature, decrease the glass transition temperature, accelerate the diffusion of a fluorescent dye through the polymer matrix and increase segmental motion. This means that the pre-exponential factor *A* (eq 3) is affected by the ionic liquid. This opens up intriguing possibilities to explore the distinct mechanisms at play in compleximers, offering new insights into their unique plasticization behavior. Through fluorescence lifetime imaging, we also find that compleximers are heterogeneous, meaning that ionic liquids may not be evenly dispersed throughout the system. We will further investigate the effect of added plasticizers, their distribution, and their influence on the relaxation dynamics and compleximer structure using molecular dynamics simulations. Additionally, it would be interesting to develop new types of compleximers, with a more flexible backbone chemistry, that can show the full thermal transition without the need of extrinsic plasticization, allowing for the characterization of pure compleximer relaxations.

## Supplementary Material



## Notes

Additional explanatary notes; additional rheological measurements; FRAP data; fluorescence lifetime distributions; dielectric spectroscopy data; tables with glass transition temperatures and apparent activation energies (PDF)
