# Supplementary material for: Unraveling the Temperature-Dependent Relaxation Dynamics of Ionic Liquid-Plasticized Compleximers
Source: Macromolecules. 2025 Jul 11;58(14):7522–33. doi: 10.1021/acs.macromol.5c01318 (PMC12288089; doi:10.1021/acs.macromol.5c01318)
Supplement: Supplementary file 1 [file ma5c01318_si_001.pdf]

Unraveling the temperature-dependent relaxation dynamics of ionic liquid-plasticized complexmers: Supporting Information

Sophie G.M. van Lange,*,† Riccardo Biella,‡ Diane W. te Brake,† Sinty Dol,† Maarten Besten,¶ Joris Sprakel,¶ Santiago J. Garcia,‡ and Jasper van der Gucht*,†

†*Physical Chemistry and Soft Matter, Wageningen University and Research, 6708 WE Wageningen, The Netherlands*

‡*Department of Aerospace Structures and Materials, Faculty of Aerospace Engineering, Delft University of Technology, 2629 HS Delft, The Netherlands*

¶*Laboratory of Biochemistry, Wageningen University and Research, 6708 WE Wageningen, The Netherlands*

E-mail: sophie.vanlange@wur.nl; jasper.vandergucht@wur.nl

Supporting Information Available

Additional notes, data, data analysis and tables.

Supporting Note S1 and S2.

Supporting Figures S1-S14.

Supporting Tables S1 and S2.

Note S1: Calculating the theoretical bond strength in compleximers

The bond energy of compleximers can be estimated using Coulomb's law:

$$U_C(r) = \frac{q_1 q_2}{4\pi\varepsilon_0\varepsilon_r r} \tag{1}$$

where r is the distance between charges, approximated here as 15 Å based on the estimated length of the hydrophobic spacers. The relative dielectric constant (ε_r) of the medium is not precisely known; however, assuming air as the medium gives a bond energy of approximately 90 kJ/mol, while using polystyrene as the medium results in a bond energy around 35 kJ/mol. These values provide an initial theoretical range for the bond strength in compleximers.

Note S2: Temperature-dependent activation energy and apparent activation energy

The activation energy E_a is commonly treated as a constant in the Arrhenius equation. However, in some systems, it may vary with temperature.[1] For instance, consider a scenario where the activation energy decreases linearly with temperature:

$$E_a = E_a(T_g) - a \cdot (T - T_g), \tag{2}$$

where $E_a(T_g)$ represents the activation energy at the glass transition temperature T_g, and a is a proportionality constant describing the rate of change of E_a with temperature. A decrease in E_a with increasing temperature could arise from thermal expansion, which increases the distances between charges and thereby weakens Coulombic interactions.

Substituting this expression into the Arrhenius equation yields:

$$\ln\left(\frac{\tau}{\tau_0}\right) = \frac{E_a(T_g) - a \cdot (T - T_g)}{RT} = -\frac{a}{R} + \frac{E_a(T_g) + a \cdot T_g}{RT}. \tag{3}$$

This equation retains a linear form when $\ln(\tau)$ is plotted against $1/T$, with the slope given by:

$$\text{slope} = \frac{E_a(T_g) + a \cdot T_g}{R}. \tag{4}$$

The apparent activation energy, E_{app}, can thus be expressed as:

$$E_{\text{app}} = E_a(T_g) + a \cdot T_g, \tag{5}$$

which is greater than the actual activation energy $E_a(T_g)$ when $a > 0$.

To illustrate this, assume that E_a decreases by 50% between T_g and $T_g + 100\,\text{K}$ (e.g., from 50 kJ/mol to 25 kJ/mol). In this case, the proportionality constant a is calculated as:

$$a = \frac{\Delta E_a}{\Delta T} = \frac{50 - 25}{100} = 0.25\,\text{kJ/mol·K}. \tag{6}$$

For a glass transition temperature of $T_g = 373\,\text{K}$, the apparent activation energy becomes:

$$E_{\text{app}} = 50 + 0.25 \cdot 373 = 145\,\text{kJ/mol}. \tag{7}$$

This value is significantly higher than the true activation energy at T_g due to the temperature dependence of E_a.

These results suggest that the high apparent activation energy often observed in such systems may be explained by a temperature-dependent E_a. Since the data retain an Arrhenius-like behavior, it is reasonable to conclude that E_a varies linearly with temperature across the studied range. Deviations from linearity in E_a would result in a non-linear Arrhenius plot, which is not observed here.

Supporting Figures

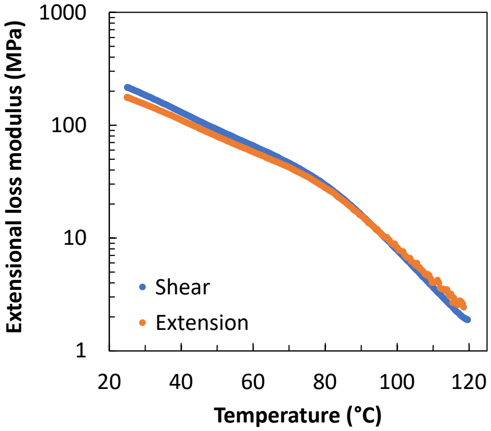

Figure S1: **Comparison of the shear storage moduli (G') and the extensional storage moduli (E')** of a temperature sweep experiment of a 35% IL-S sample performed at 1 Hz. The extensional moduli were converted to shear moduli assuming a Poisson ratio of 0.5, so that E=3G. The good overlap indicates the absence of compliance issues.

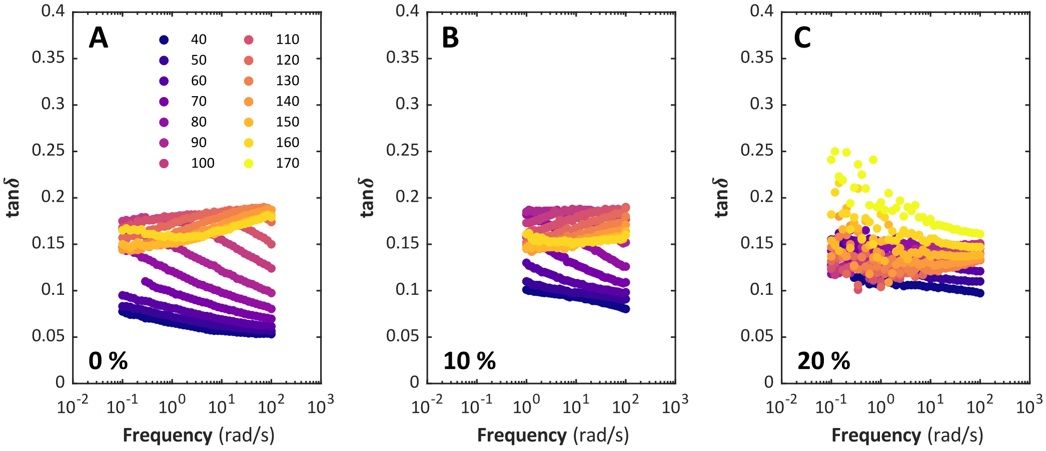

Figure S2: **The unshifted tanδ values corresponding to the TTS results of compleximers with: (A)**, 0% IL-S. **(B)**, 10% IL-S. **(C)**, 20% IL-S.

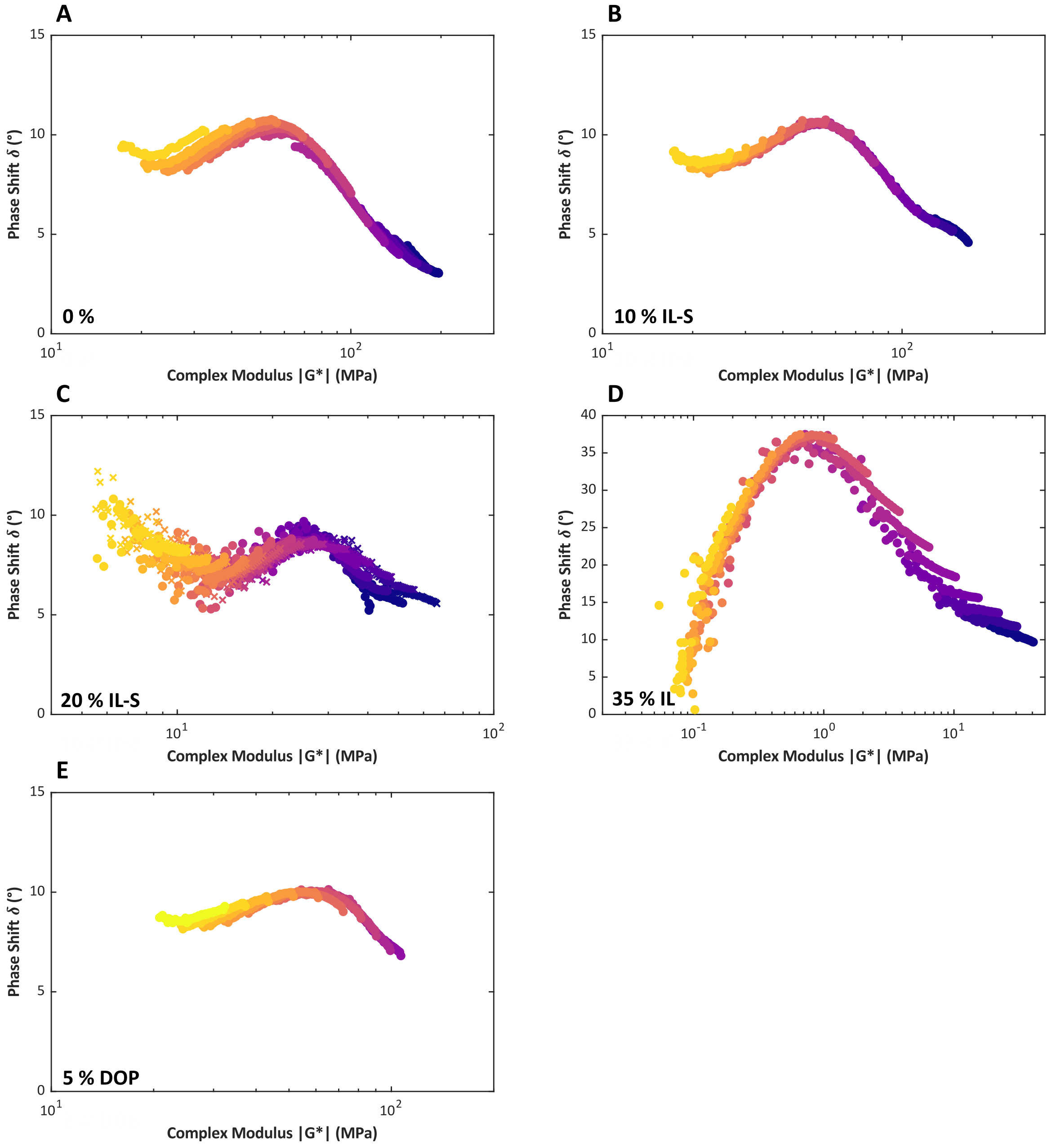

Figure S3: **The Van Gurp-Palmen plots of compleximers plasticized with (A)**, 0% ionic liquid. **(B)**, 10% IL-S. **(C)**, 20% IL-S. **(D)**, 35% IL. **(E)**, 5% DOP. Isothermal frequency curves are expected to collapse onto a single smooth line when time–temperature superposition (TTS) is applicable.[2] Overall, TTS holds well for samples with low plasticizer concentrations. At higher concentrations the plots become more scattered, possibly indicating a (small) deviation from rheological simplicity.

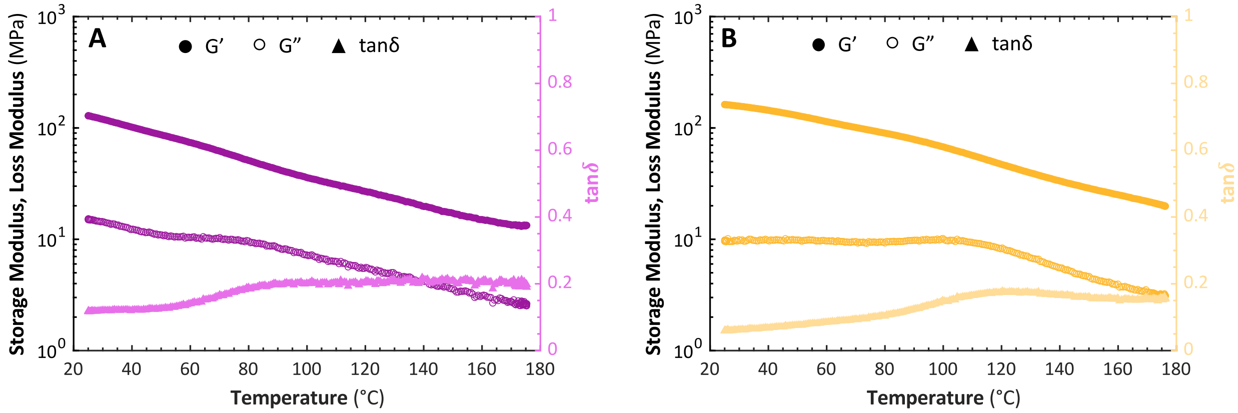

Figure S4: **Temperature sweeps of compleximers plasticized with (A)**, 10% IL-S. **(B)**, 5% DOP.

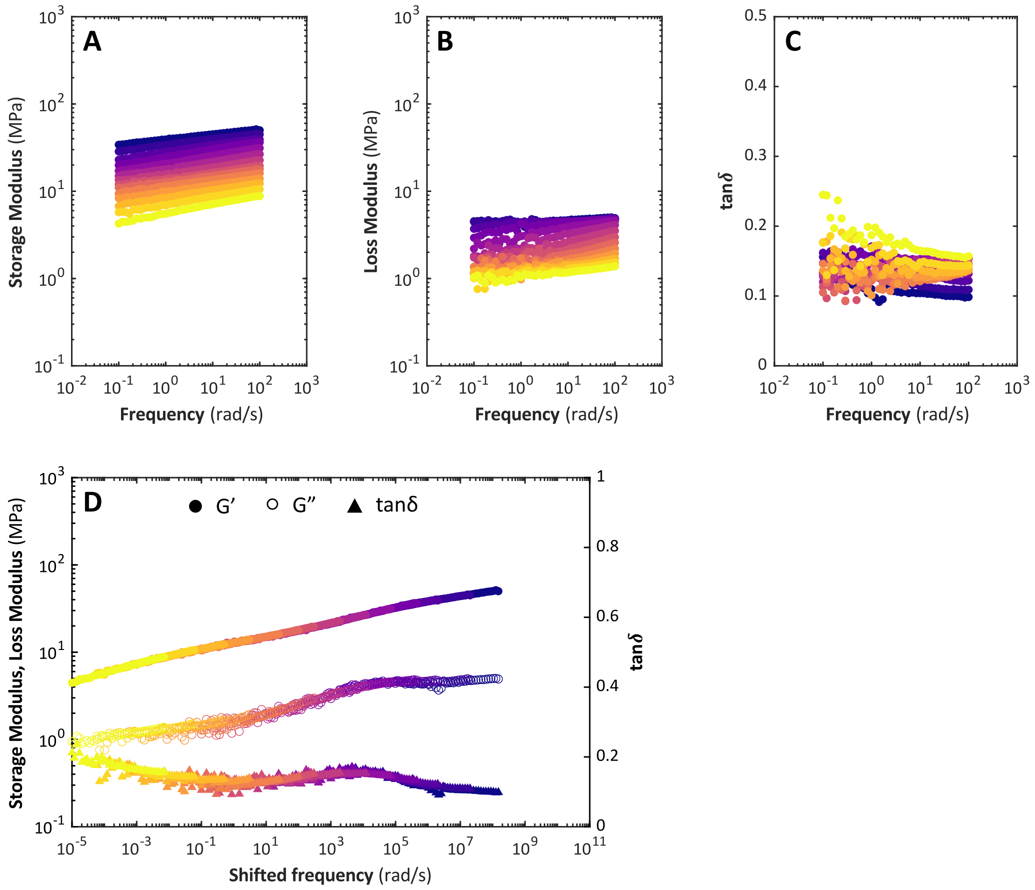

Figure S5: **TTS figures of the duplicate measurement of compleximer with 20% IL-S. (A)**, Storage modulus. **(B)**, Loss modulus. **(C)**, tanδ. **(D)**, Master curve.

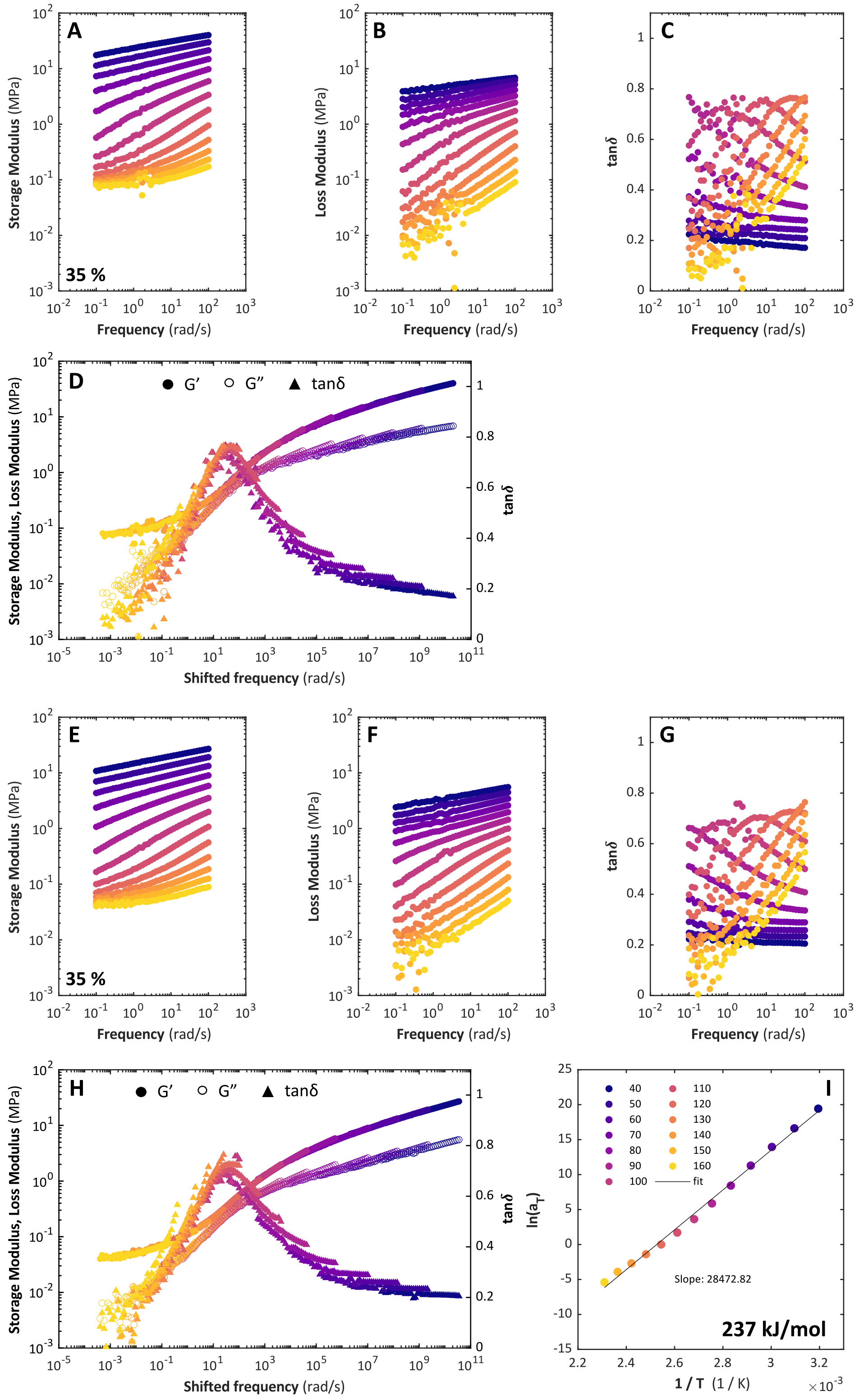

Figure S6: **TTS figures of two compleximer samples plasticized with 35% IL. (A)**, Storage modulus. **(B)**, Loss modulus. **(C)**, tanδ. **(D)**, Master curve. **(E)**, Storage modulus. **(F)**, Loss modulus. **(G)**, tanδ. **(H)**, Master curve. **(I)**, Arrhenius plot and activation energy.

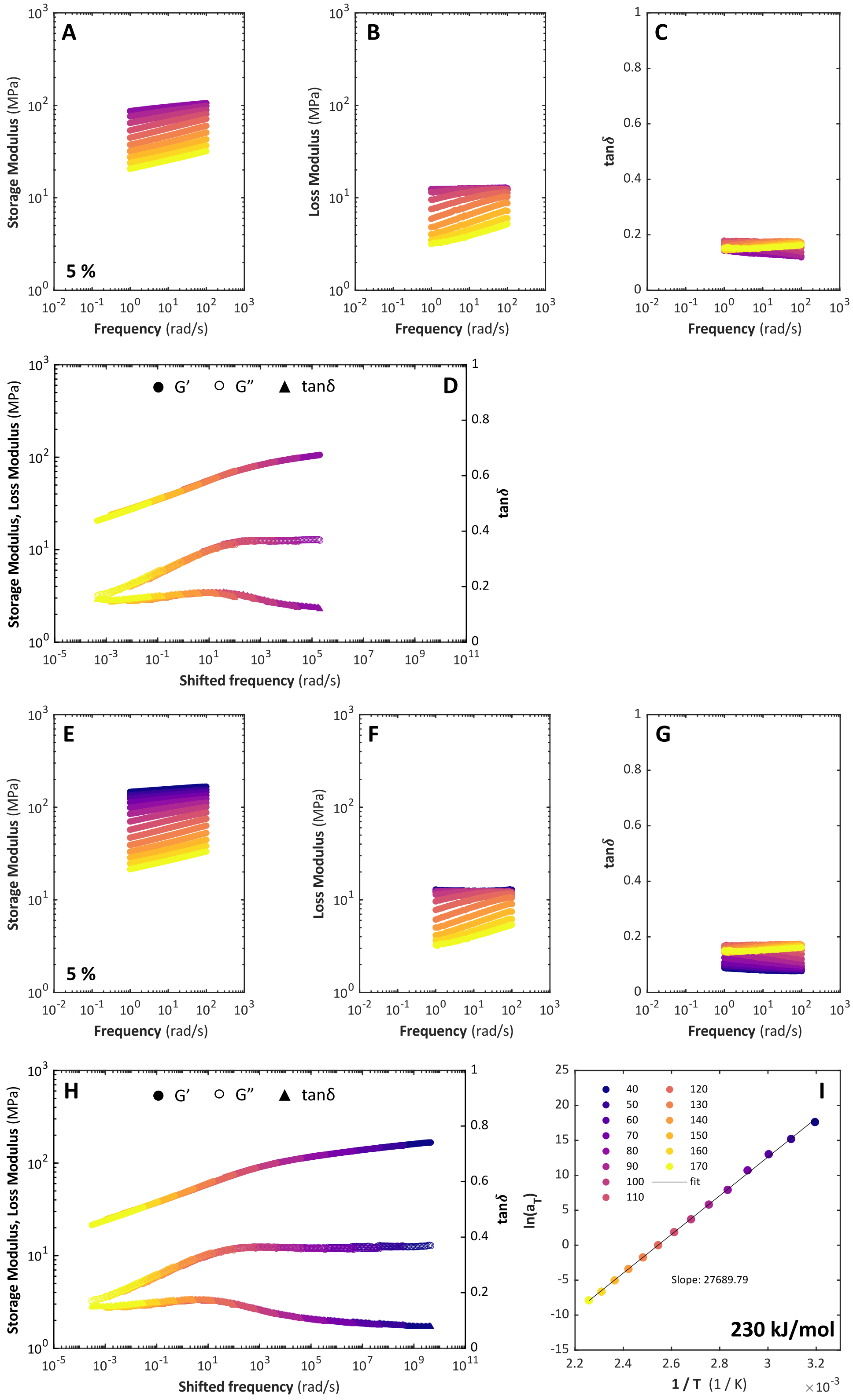

Figure S7: **TTS figures of two compleximer samples plasticized with 5% DOP. (A)**, Storage modulus. **(B)**, Loss modulus. **(C)**, tanδ. **(D)**, Master curve. **(E)**, Storage modulus. **(F)**, Loss modulus. **(G)**, tanδ. **(H)**, Master curve. **(I)**, Arrhenius plot and activation energy.

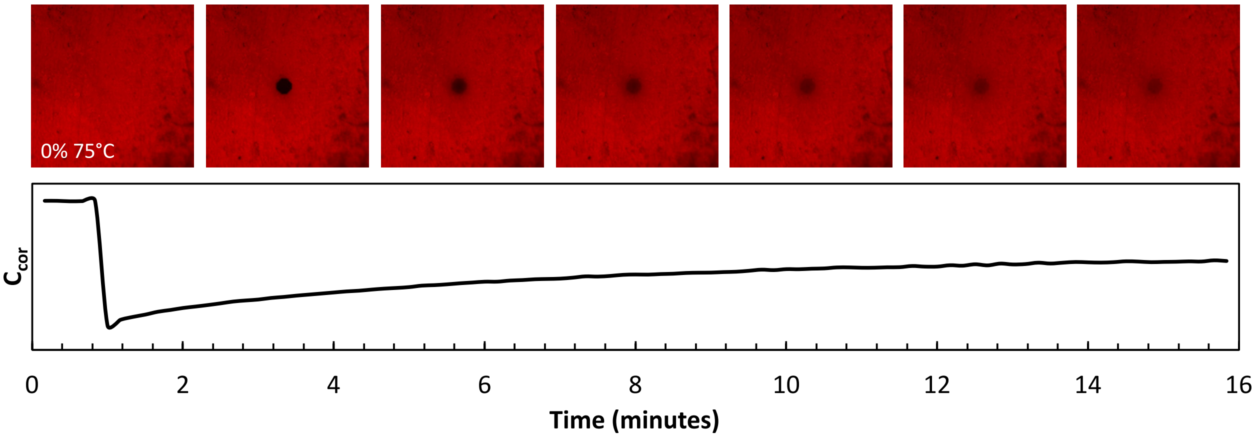

Figure S8: **Example of a FRAP experiment.** A region of interest (ROI) is bleached with a high intensity laser pulse. The fluorescence recovery is monitored over the course of 15 minutes, during which the fluorescent dye diffuses from the surrounding region back to the region of interest. The average intensity in the ROI is recorded in every frame, and corrected with the average intensity in a reference spot far away from the bleached region. The corrected recovery was plotted as a function of time.

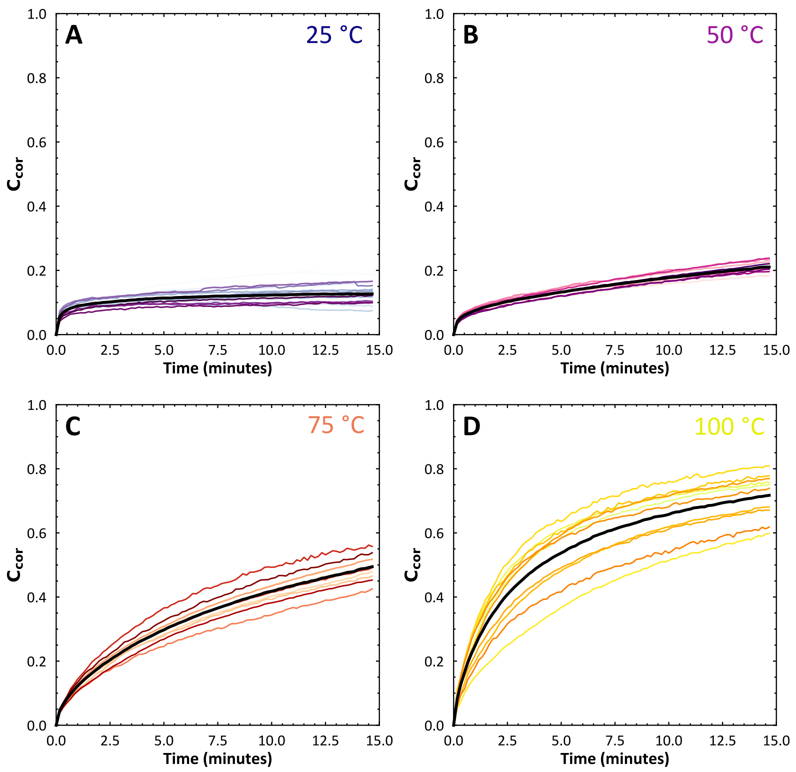

Figure S9: **Recovery curves of non-plasticized compleximer at four different temperatures: (A)**, 25 °C. **(B)**, 50 °C. **(C)**, 75 °C. **(D)**, 100 °C. The average is plotted in black.

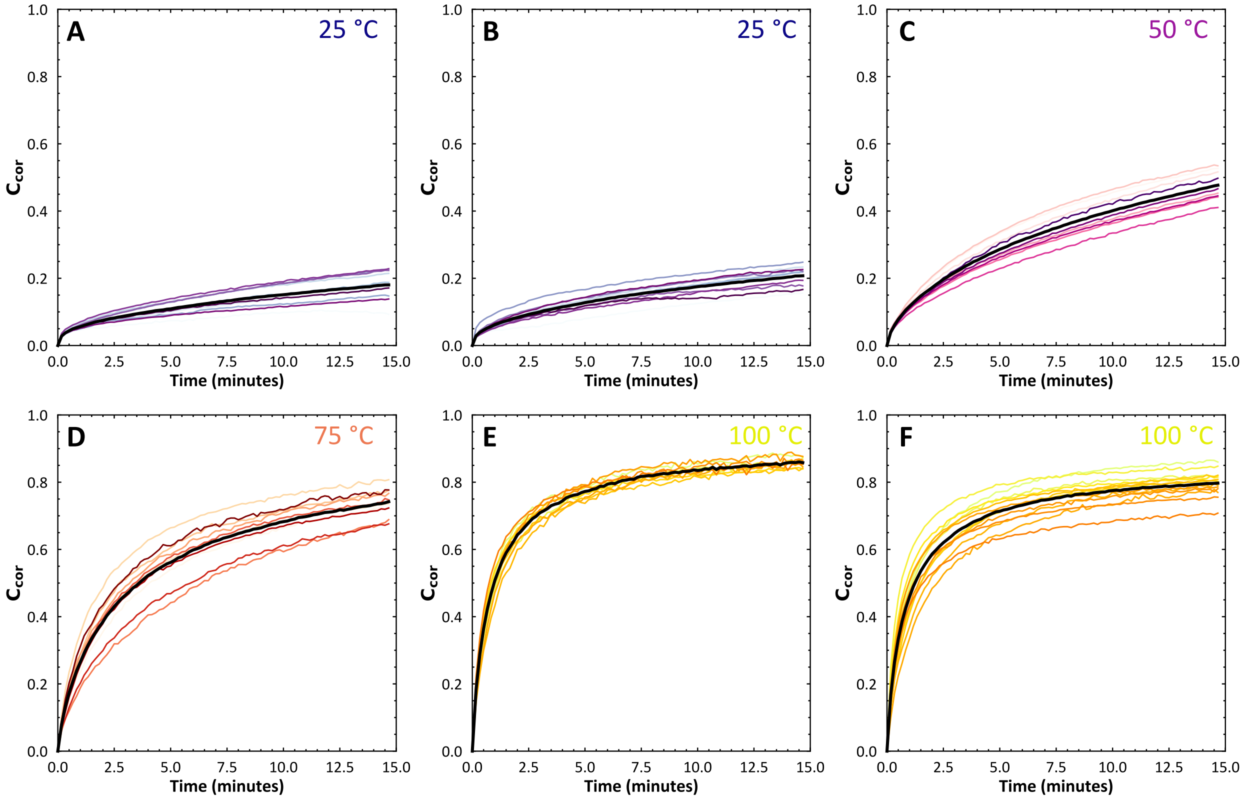

Figure S10: **Recovery curves of compleximer plasticized with 20% IL-S at four different temperatures: (A)**, 25 °C. **(B)**, duplicate measurement of 25 °C. **(C)**, 50 °C. **(D)**, 75 °C. **(D)**, 100 °C. **(D)**, duplicate measurement of 100 °C. The average is plotted in black.

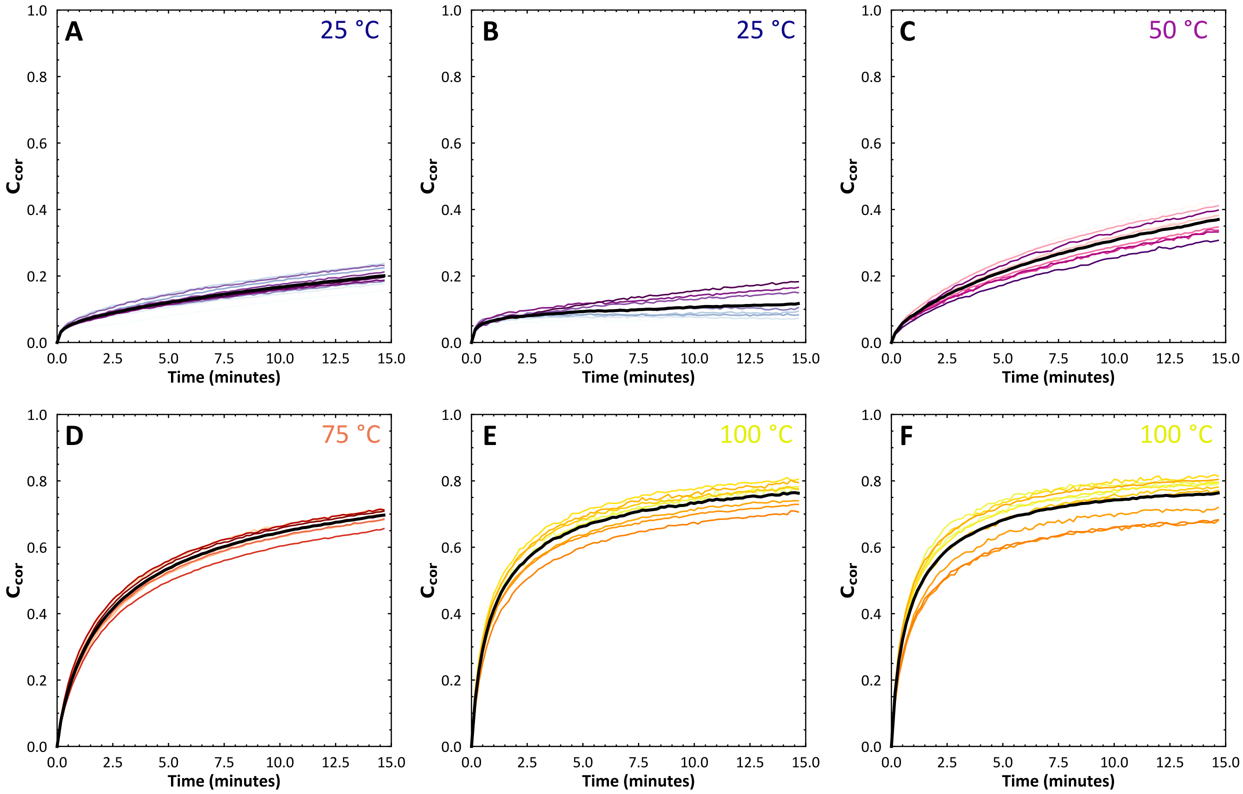

Figure S11: **Recovery curves of complexımer plasticized with 35% IL at four different temperatures: (A)**, 25 °C. **(B)**, duplicate measurement of 25 °C. **(C)**, 50 °C. **(D)**, 75 °C. **(D)**, 100 °C. **(D)**, duplicate measurement of 100 °C. The average is plotted in black.

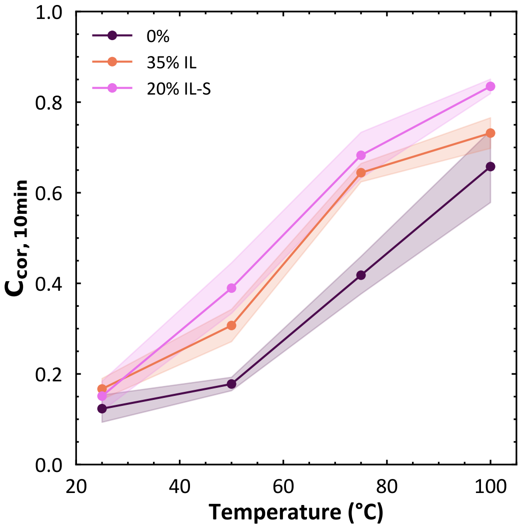

Figure S12: **The average corrected intensity at 10 minutes after bleaching** was plotted as a function of temperature. The recovery increases with temperature, and the screened ionic liquid IL-S is slightly more effective than IL.

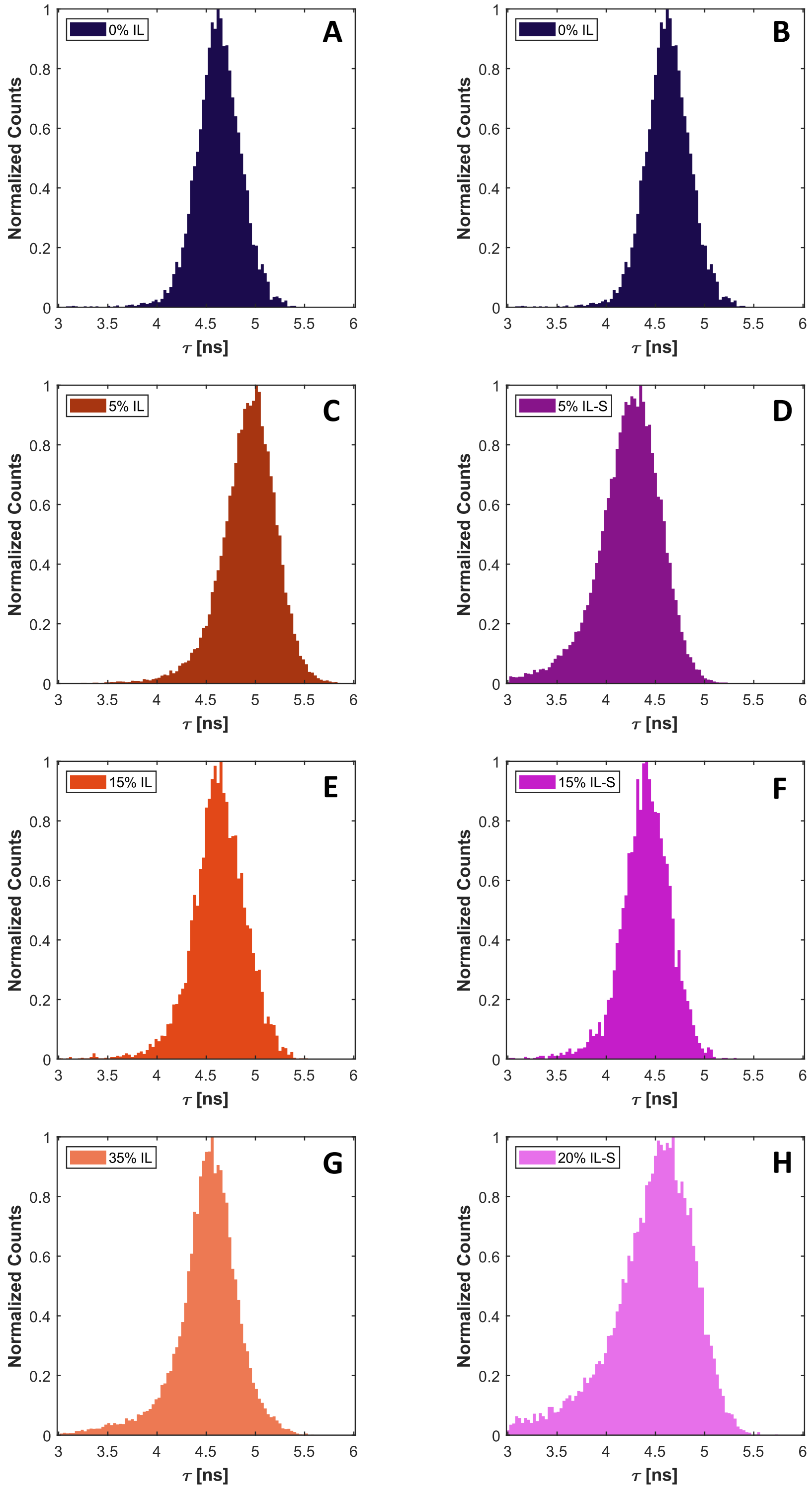

Figure S13: **The lifetime distribution histograms of plasticized compleximers with: (A,B)**, 0% ionic liquid. **(C)**, 5% IL. **(D)**, 5% IL-S. **(E)**, 15 % IL. **(F)**, 15 % IL-S. **(G)**, 35% IL. **(H)**, 20 % IL-S.

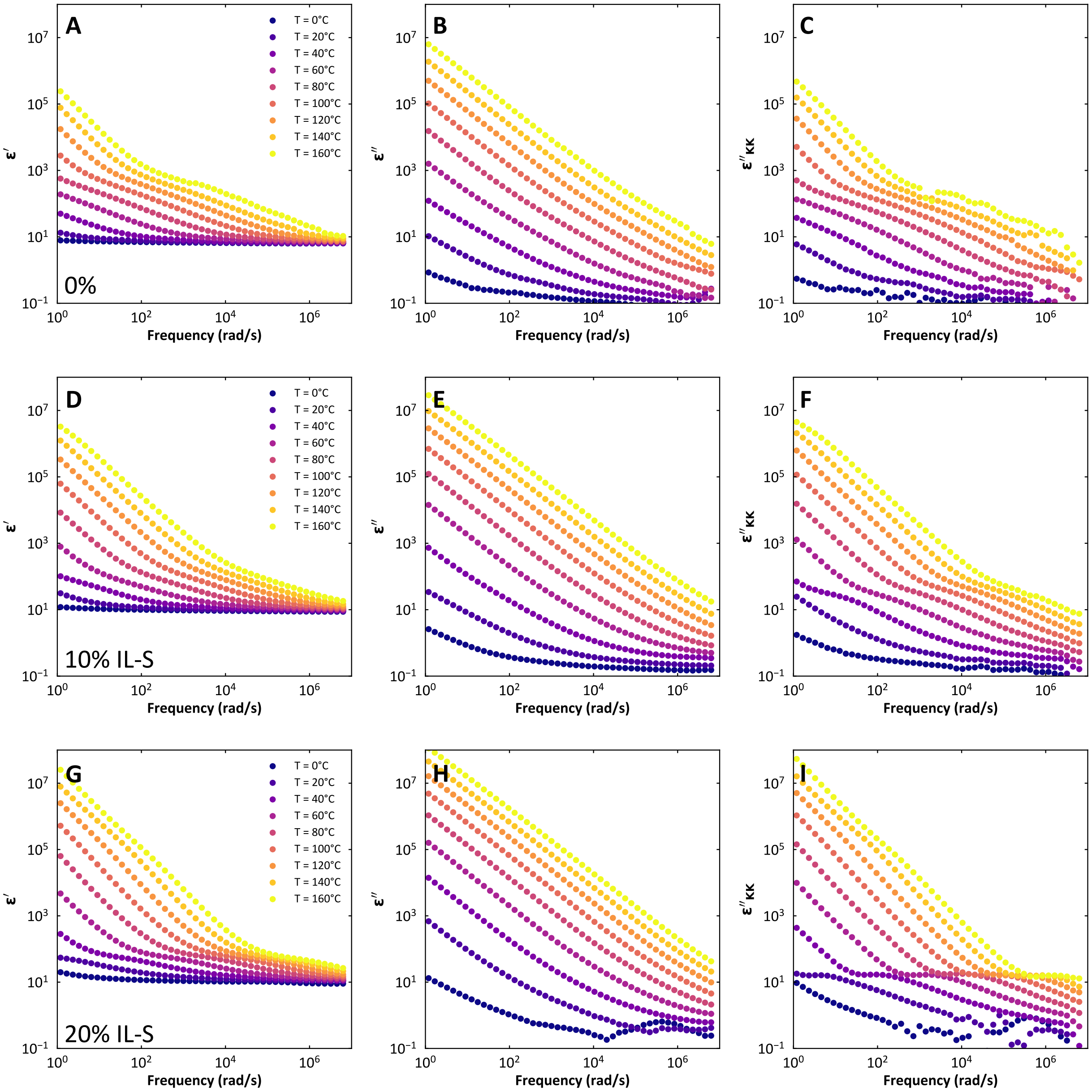

Figure S14: **The real ε', imaginary ε'' and Kramers-Kronig-transformed $\varepsilon''_{\mathbf{KK}}$ part of the dielectric permittivity of samples with: (A-C)**, 0%, **(D-F)**, 10% **(C-I)**, and 20% IL-S.

Supporting Tables

Table S1: **Glass transition temperature of plasticized compleximers.** The T_g of the non-plasticized compleximer was outside the measurement window. The T_g was determined by finding the peak in the tanδ in the temperature sweep.[3]

Material	Glass transition temperature T_g (°C)
S compleximer	-
S compleximer 10% IL-S	95
S compleximer 20% IL-S	84
S compleximer 35% IL	140
S compleximer 5% DOP	125

Table S2: **Activation energies of compleximers** from rheological Time Temperature Superposition (TTS), fluorescence recovery after photobleaching (FRAP) and broadband dielectric spectroscopy (BDS) TTS. Values that contain data below T_g are indicated with an asterisk *.

**Material**	**Rheology** (kJ/mol)	**FRAP** (kJ/mol)	**BDS** (kJ/mol)
**S compleximer**	252*	57*	114*
**S compleximer 10% IL-S**	192		82.5
**S compleximer 20% IL-S**	235	54*	78.4
**S compleximer 35% IL**	176	47*	
**S compleximer 5% DOP**	186		

References

(1) Struik, L. The apparent activation energy for mechanical and dielectric relaxation in glass-forming (polymeric) liquids: A misconception? *Polymer* **1997**, *38*, 733–735.

(2) Trinkle, S.; Friedrich, C. Van Gurp-Palmen-plot: a way to characterize polydispersity of linear polymers. *Rheologica Acta* **2001**, *40*, 322–328.

(3) van Lange, S. G. M.; Te Brake, D. W.; Portale, G.; Palanisamy, A.; Sprakel, J.; van der Gucht, J. Moderated ionic bonding for water-free recyclable polyelectrolyte complex materials. *Science Advances* **2024**, *10*, eadi3606.
